# Early Rise of Blood T Follicular Helper Cell Subsets and Baseline Immunity as Predictors of Persisting Late Functional Antibody Responses to Vaccination in Humans

**DOI:** 10.1371/journal.pone.0157066

**Published:** 2016-06-23

**Authors:** Fabiana Spensieri, Emilio Siena, Erica Borgogni, Luisanna Zedda, Rocco Cantisani, Nico Chiappini, Francesca Schiavetti, Domenico Rosa, Flora Castellino, Emanuele Montomoli, Caroline L. Bodinham, David J. Lewis, Duccio Medini, Sylvie Bertholet, Giuseppe Del Giudice

**Affiliations:** 1 Novartis Vaccines & Diagnostics S.r.l., Siena, Italy; 2 Departement of Molecular and Developmental Medicine, University of Siena, & VisMederi S.r.l., Siena, Italy; 3 Surrey Clinical Research Center, University of Surrey, Guildford, United Kingdom; Saint Louis University Division of Infectious Diseases and Immunology, UNITED STATES

## Abstract

CD4^+^ T follicular helper cells (T_FH_) have been identified as the T-cell subset specialized in providing help to B cells for optimal activation and production of high affinity antibody. We recently demonstrated that the expansion of peripheral blood influenza-specific CD4^+^IL-21^+^ICOS1^+^ T helper (T_H_) cells, three weeks after vaccination, associated with and predicted the rise of protective neutralizing antibodies to avian H5N1. In this study, healthy adults were vaccinated with plain seasonal trivalent inactivated influenza vaccine (TIIV), MF59^®^-adjuvanted TIIV (ATIIV), or saline placebo. Frequencies of circulating CD4^+^ T_FH_1 ICOS^+^ T_FH_ cells and H1N1-specific CD4^+^IL-21^+^ICOS^+^ CXCR5^+^ T_FH_ and CXCR5^-^ T_H_ cell subsets were determined at various time points after vaccination and were then correlated with hemagglutination inhibition (HI) titers. All three CD4+ T cell subsets expanded in response to TIIV and ATIIV, and peaked 7 days after vaccination. To demonstrate that these T_FH_ cell subsets correlated with functional antibody titers, we defined an alternative endpoint metric, decorrelated HI (DHI), which removed any correlation between day 28/day 168 and day 0 HI titers, to control for the effect of preexisting immunity to influenza vaccine strains. The numbers of total circulating CD4^+^ T_FH_1 ICOS^+^ cells and of H1N1-specific CD4^+^IL-21^+^ICOS^+^ CXCR5^+^, measured at day 7, were significantly associated with day 28, and day 28 and 168 DHI titers, respectively. Altogether, our results show that CD4^+^ T_FH_ subsets may represent valuable biomarkers of vaccine-induced long-term functional immunity.

**Trial Registration:**

ClinicalTrials.gov NCT01771367

## Introduction

Protein-based vaccines confer protection against pathogens mainly through the induction of T cell-dependent high affinity functional antibody responses. In this context a specialized subset of T helper cells (T_H_), identified as T follicular helper cells (T_FH_), differentiate and provide help to B cells in the germinal centers (GC) of secondary lymphoid organs, leading to B-cell proliferation and differentiation, and reshaping of the B-cell repertoire and Ig affinity maturation [1–5]. Thus, T_FH_ cells play a critical role in the generation of long-lived humoral responses to antigens [3].

T_FH_ cells were first isolated and identified in human tonsils, and were characterized by the expression of B cell follicle homing chemokine receptor CXCR5 and the inducible costimulatory molecule ICOS [6, 7]. T_FH_ cells efficiently provide help to B cells and promote IgM to IgG immunoglobulin class switching through the production of interleukin-21 (IL-21) [8]. Studies in animal models have shown that, once differentiated and activated, T_FH_ cells can exit GC, developing into memory T_FH_ cells [9–12]. However, the origin of human blood circulating T_FH_ cells remains to be established.

CD4^+^ T_H_ cells expressing the chemokine receptor CXCR5 are currently termed blood memory or peripheral T_FH_ cells and are long-lived memory cells [7, 13–15]. Recently, some human studies have contributed to a deeper characterization of blood T_FH_ cells on the basis of the expression of additional chemokine receptors such as CXCR3, CCR6, and CCR7, the costimulatory molecule ICOS, and the immunomodulatory molecule PD-1 [13, 16, 17]. T_FH_ cells defined as CXCR3^+^CCR6^-^ share properties with T_H_1 cells (hereafter called T_FH_1 cells), while CXCR3^-^CCR6^-^ and CXCR3^-^CCR6^+^ cells share properties of T_H_2 cells (T_FH_2) and of T_H_17 cells (T_FH_17), respectively [13]. T_FH_2 and T_FH_17 have a more efficient T helper activity on naive B cells, while T_FH_1 ICOS^+^ cells have a higher propensity to provide help to memory B cells [17]. In addition, we previously demonstrated that antigen-specific T_FH_ can be identified by flow cytometry by intracelluar staining of IL-21 upon *in vitro* antigen stimulation [18].

The identification of early biomarkers predicting vaccine efficacy may contribute to accelerate the development of novel vaccine candidates. These biomarkers should be easy to test in large clinical trials and have a clear mechanistic relationship with the correlates or surrogates of protection taken as study’s endpoints. Recent studies showed that immunization with influenza A/California/2009 (H1N1) vaccine led to an expansion of peripheral T_FH_ subsets in humans [13, 17, 19–21]. Moreover, *ex vivo* frequencies of peripheral T_FH_1 cells at day 7 correlated with the frequency of circulating plasmablasts and with increased levels of neutralizing antibodies to H1N1 at day 21 [13, 17]. In a previous study, we showed that a single dose of an avian H5N1 influenza vaccine induced the expansion of H5N1-specific CD4^+^ICOS^+^IL-21^+^ T_H_ cells in the blood three weeks after vaccination, and that the increased frequency of these cells predicted the protective antibody titers found after the second dose of the vaccine [18].

The goal of the present study was to identify, in human peripheral blood, early T_FH_ cells subset(s) predicting not only the rise but also the long term persistence of functional antibody titers after seasonal influenza vaccination. For this purpose, we had access to human PBMCs collected in the framework of the European Innovative Medicine Initiative funded public-private project BIOVACSAFE [22]. PBMCs from healthy subjects immunized with one dose of seasonal adjuvanted or non-adjuvanted trivalent inactivated influenza vaccine (ATIIV and TIIV, respectively) were analyzed both directly *ex vivo* or after *in vitro* antigen stimulation. Frequencies of T_FH_ cells were determined and then correlated with HI antibody titers measured at days 28 and 168 post-vaccination. Both antigen-specific CD4^+^IL-21^+^ICOS^+^CXCR5^+^ T_FH_ cells and *ex vivo* T_FH_1 ICOS^+^ cells expanded seven days after vaccination and returned to baseline levels by day 28. After accounting for the effect of baseline HI titers, we showed that the magnitude of the response of these T_FH_ cell subsets correlated with functional antibody responses measured up to 6 months after vaccination.

## Materials and Methods

### Clinical samples and vaccines

**Fig 1:**
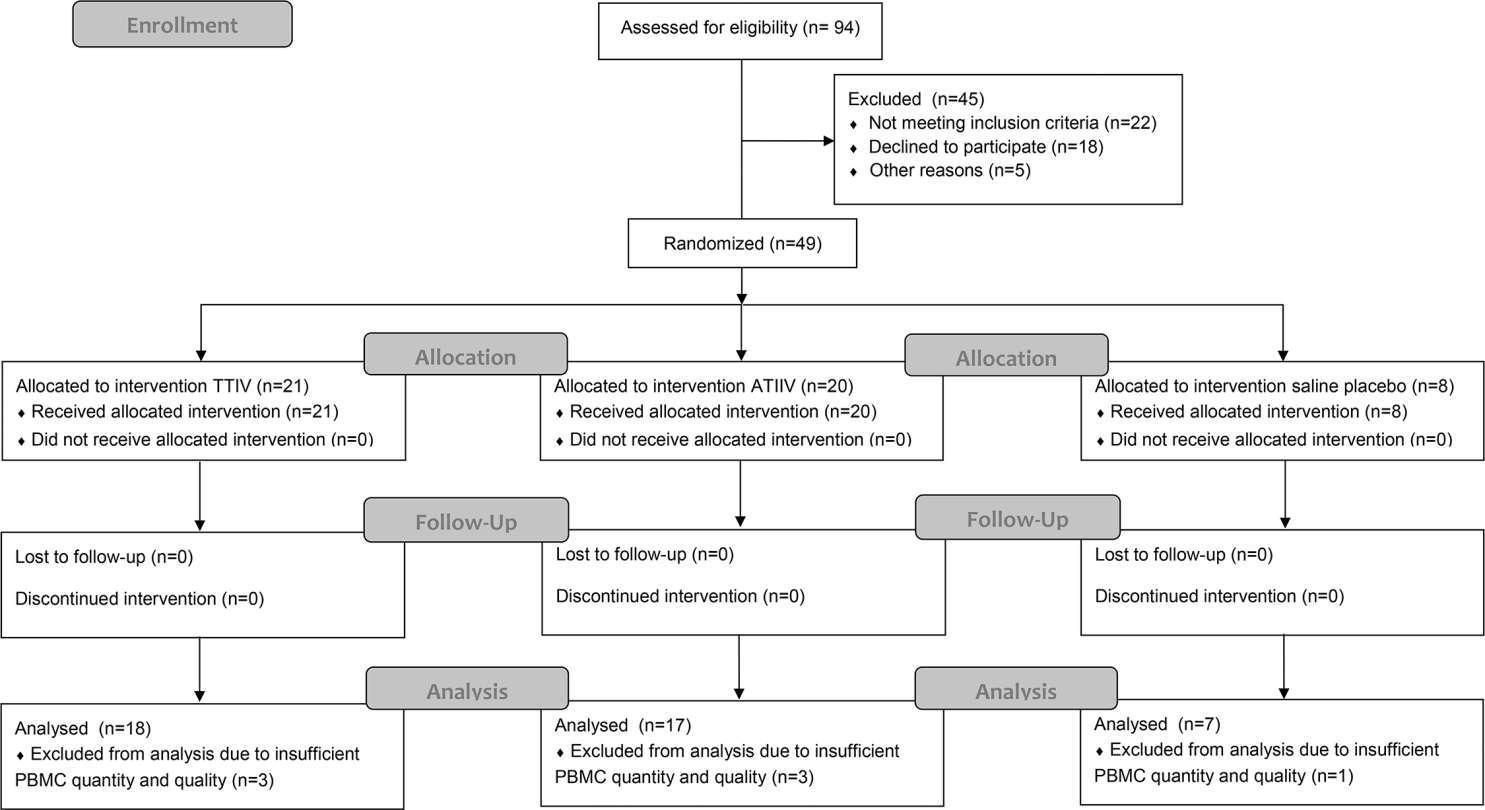
CONSORT Flow diagram.

The study received ethical approval from London—Surrey Borders Research Ethics Committee (REC Ref: 13/LO/0044), and was registered on ClinicalTrials.gov prior to enrolment (NCT01771367). Forty-nine healthy adults (18–43 years old) were enrolled at the Surrey Clinical Research Centre, University of Surrey, Guildford, UK, as part of the BIOVACSAFE Consortium-funded clinical trial protocol CRC305C sponsored by the University of Surrey. The study was a partial-blind (participant and laboratory), randomised, placebo controlled exploratory study. The full study protocol is described in supplementary document S1 Text. All participants provided written informed consent. In the 2012-2013 winter season, 49 participants were randomized to allow 48 to complete with the full required reportable data in three arms and received one dose of A/California/7/2009 (H1N1), A/Victoria/361/2011 (H3N2), B/Wisconsin/1/2010-like TIIV (Agrippal^®^; n=21), ATIIV (Fluad^®^, n=20), or saline placebo (n=8). Recruitment for the study began on February 7^th^ 2013 and ended with the last follow-up visit on November 25^th^ 2013. See CONSORT diagram and checklist (Fig 1 and S2 Text, respectively). PBMCs were collected from each group at baseline, day 7 and day 28 after immunization, and analyzed for plasmablasts and CD4^+^ T cell responses. Sera were collected at day 0, day 7, day 28 and day 168 after immunization for the analyses of antibody responses.

### Hemagglutination Inhibition Assay (HI)

HI titers were measured for A/California/7/2009 (H1N1), A/Victoria/361/2011 (H3N2), and B/Wisconsin/1/2010-like vaccine strains (Novartis Vaccines & Diagnostics) as described elsewhere [23, 24].

### Polychromatic flow cytometry analyses

Frozen PBMCs from vaccinated participants were thawed and then analyzed by flow cytometry both *ex vivo* and after 18 h of stimulation at 37°C with anti-CD28 and anti-CD49d (1 μg/ml each, BD Biosciences), A/California/7/2009 (H1N1) subunit vaccine antigen (1 μg/ml, Novartis Vaccines & Diagnostics), or *Staphylococcus* enterotoxin B (SEB) (1 μg/ml, Sigma), in the presence of Brefeldin A (5 μg/ml, Sigma) as previously described [18, 25]. Cells were stained *ex vivo* with Live/Dead Yellow (Invitrogen), fluorochrome-conjugated antibodies: CD3-PE-Texas Red (SK7), CD4-APC-Horizon 7 (SK3), ICOS-PE (ISA-3), CXCR5-FITC (RF8B2), CXCR3-PE-Cy5 (1C6), CCR6-Brillant Violet 421 (11A9), PD1-Brilliant Violet 785 (EH12.2H7), CD19-APC (SJ25C1), CD20-PerCP-Cy5.5 (L27), CD27-PE (L128), CD38-Alexa fluor 700 (HIT2) (BD Biosciences), CD8-Horizon V500 (RPA-T8) (Biolegend), CD45RA-PE-Cy7 (HI100) (eBioscience). H1N1-specific CD4^+^ T cells were analyzed for intracellular production of IL-21 with anti-IL-21-APC (3A3-N2.1) (BD Biosciences) [18, 25]. Stained cells were acquired on a BD LSR Fortessa special order flow cytometer (BD Biosciences).

### Analysis of H1N1-specific CD4^+^IL-21^+^ ICOS^+^ T_H_ cells

Frequency, phenotype, and cytokine profile of H1N1-specific CD4^+^IL-21^+^ICOS^+^ T_H_ cells were determined by polychromatic flow cytometry following the gating strategy described in S1C Fig. The response to medium was subtracted for each subject at each time point. Data are expressed as number of antigen-specific CD4^+^ T cells per million of total CD4^+^ T cells. Data were analyzed using FlowJo (version 9.6, Tree Star) [18].

### Statistical analyses

Median HI titers and frequencies of CD4^+^ T cells measured across different time points were compared using Wilcoxon’s signed rank test. Analyses were performed with SAS JMP 8.0.1 software. Associations between day 7 cellular responses and day 28 and 168 functional antibody responses were evaluated using the Pearson product-moment correlation metric. Data from TIIV and ATIIV vaccine cohorts were merged and analyzed as a single dataset. The potential effect of vaccine formulation as well as the negative correlation between HI responses to vaccination and baseline titers were removed, for each antigen, by fitting a mixed model and obtaining the residuals, based on a method adapted from Bucasas *et al*. [26]. The R package *lme4* was used to perform a linear mixed effects analysis of the relationship between day 28, or day 168, HI titers fold-increase and baseline HI titers [27]. The baseline HI titer was used as only fixed effect, while random slopes and intercepts for the two vaccine cohorts were included as random effects. The model was formulated as follows: HI_DayX/Day0_ ~ HI_Day0_ + (1 + HI_Day0_|Cohort) + ε. Where HI_DayX/Day0_ represents the HI fold-increase measured either at day 28 or 168 post-vaccination, HI_Day0_ corresponds to the baseline HI titer, Cohort is a 2-level factor indicating which vaccine was used and ε is the residual error. Given the lack of antigen specificity, cell responses derived from the *ex vivo* staining were correlated to the maximun response observed across the three strains represented in the vaccine, while H1N1-specific T_FH_ cells were correlated to H1N1-specific HI responses.

## Results

Of the participants who were randomised, PBMCs of sufficient quantity and quality were obtained for 18 participants who received TIIV, 17 participants who received ATIIV and 7 who received placebo. Therefore the results reported below are only for 42 participants.

### Influenza vaccination induces fast and long-lasting functional antibody responses

In this study, 42 healthy adults received a single intramuscular immunization with 2012-2013 TIIV, ATIIV, or saline placebo. B-cell responses were characterized as (i) frequencies of blood CD19^+^CD20^-^CD38^+^ plasmablasts measured by flow cytometry at day 0, 7 and 28, and (ii) as vaccine-specific HI antibody titers at day 0, 7, 28, and 168.

**Fig 2:**
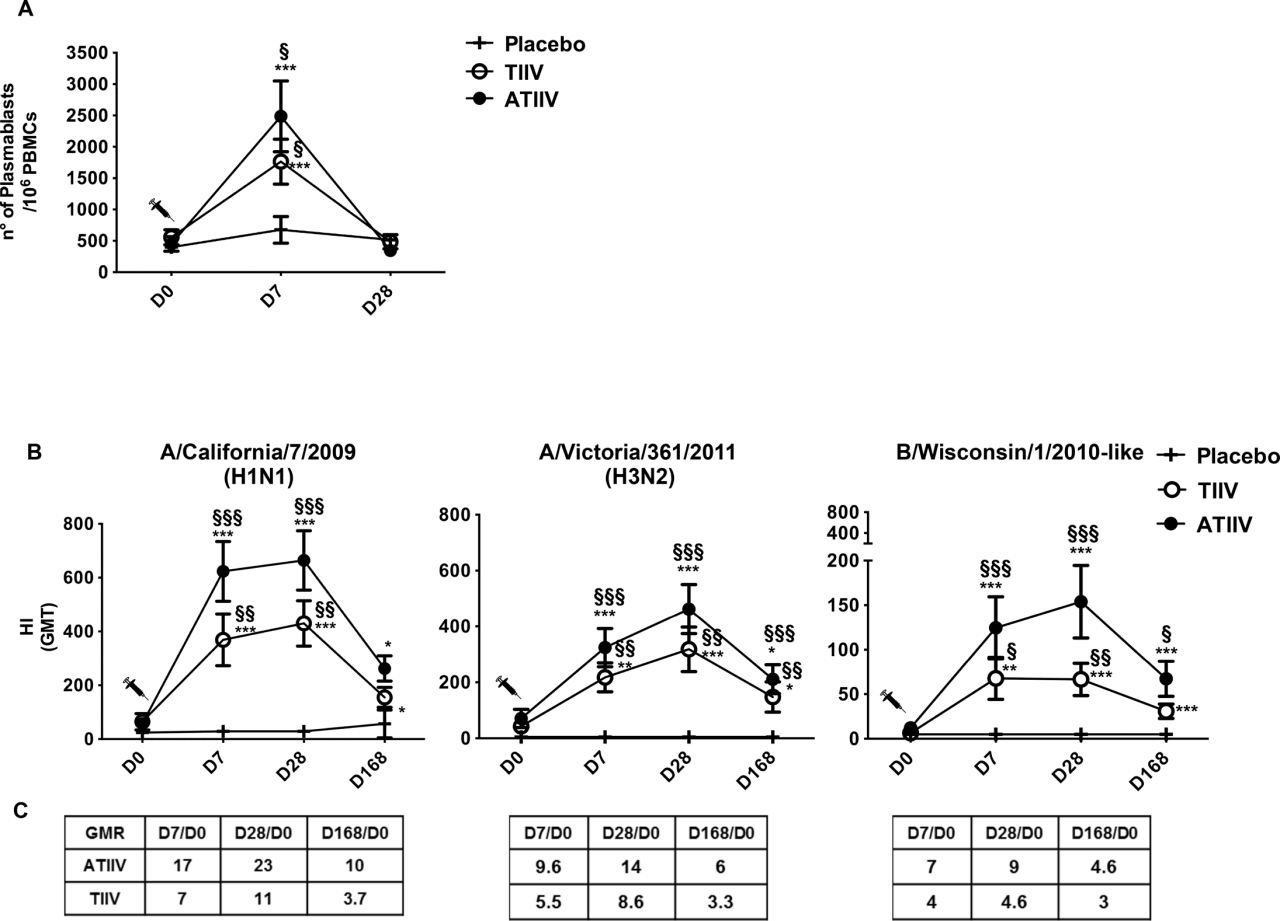
B-cell and functional antibody responses after seasonal influenza vaccination. (**A**) Absolute number of plasmablasts (CD19^+^CD20^-^ CD38^+^) in 10^6^ live PBMCs acquired. (**B**) HI Geometric mean titers (GMT) for A H1N1 and H3N2, and B influenza strains at baseline (D0), 7 days (D7), 28 days (D28), and 168 days (D168) after a single dose of influenza vaccine. Data show three cohorts: saline placebo (n=7), TIIV (n=18), and ATIIV (n=17). (**C**) Geometric mean ratio (GMR) for all vaccine strains. Non-parametric Wilcoxon’s signed rank test was used for statistical analyses: *p < 0.05, **p < 0.01, and ***p < 0.001 compared to day 0; ^§^*p* < 0.05, ^§§^*p* < 0.01 and ^§§§^*p* < 0.001 compared to saline placebo.

Consistent with previous studies [28–30], TIIV and ATIIV increased the frequencies of plasmablasts at day 7 compared to placebo (Fig 2A and S1A Fig for subset identification). HI geometric mean antibody titers (GMT) against the three influenza virus strains significantly increased 7 days after immunization compared to placebo. HI antibody titers peaked at day 28, and peristed up to six months later at titers which were significantly higher than placebo for the H3N2 and B strains following ATIIV (Fig 2B and 2C). These higher titers at 6 months post-ATIIV administration paralleled seroprotection rates which were higher after ATIIV than TIIV (S1 Table).

### Circulating T_FH_1 cells expressing ICOS expand after both TIIV and ATIIV influenza vaccination

**Fig 3:**
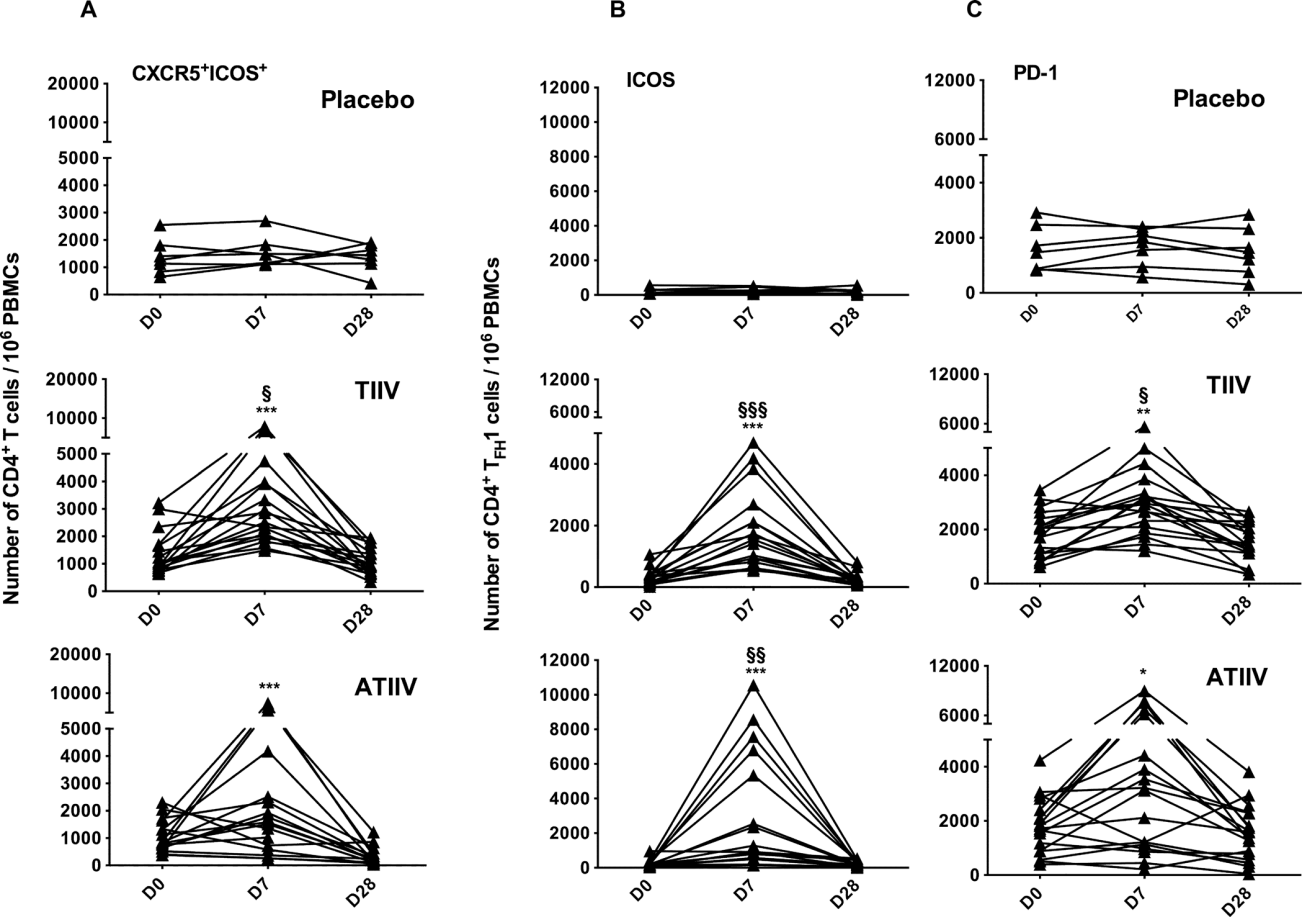
Expansion of ICOS^+^ and PD-1^+^ T_FH_1 cells after TIIV and ATIIV vaccination. (**A**) Number of CD4^+^ T cells expressing CXCR5 and ICOS in human PBMCs after seasonal influenza vaccination. (**B** and **C**) Number of T_FH_1 cells expressing ICOS and PD-1. Data show three cohorts: saline placebo (n=7), TIIV (n=18) and ATIIV (n=17) at baseline (D0), day 7 (D7) and day 28 (D28) after a single dose of influenza vaccine. Data are shown for each participant and expressed as number of cells in 10^6^ live PBMCs acquired. Non-parametric Wilcoxon’s signed rank test was used for statistical analyses: *p < 0.05, **p < 0.01, and ***p < 0.001 compared to day 0; ^§^*p* < 0.05, ^§§^*p* < 0.01 and ^§§§^*p* < 0.001 compared to saline placebo.

The number of CD4^+^ICOS^+^ T_H_ cells expressing CXCR5 (CXCR5^+^ICOS^+^) increased at day 7 after vaccination with TIIV and ATIIV and returned to baseline levels by day 28 (Fig 3A). Vaccination did not affect the number of CXCR5^+^ICOS^-^ nor of CXCR5^-^ICOS^+^ CD4^+^ T_H_ cells (S2 Fig), in agreement with previous observations. [17]. We then determined the expression of ICOS in different T_FH_ subsets in PBMC at day 0, 7, and 28 after administration of TIIV or ATIIV (S1B Fig for subset identification) [17]. The number of CD4^+^ T_FH_1 ICOS^+^ and PD-1^+^ cells peaked at day 7 after vaccination with TIIV and ATIIV compared to day 0, returned to baseline levels by day 28, and were also significantly higher compared to saline placebo controls (Fig 3B and 3C). Vaccination did not modify the frequencies of ICOS^+^ cells in the T_FH_17 and T_FH_2 cell subsets (S1B and S3 Figs). No changes in numbers of CD4^+^ T_FH_ cells were observed in the placebo controls at any time point. Thus, these data show that peripheral blood T_FH_1 cells, expressing ICOS and PD-1, transiently expand at day 7 after TIIV, as shown previously [17], and also after ATIIV.

### Antigen-specific CD4^+^IL-21^+^ICOS^+^ T_H_ cells expand at day 7 after vaccination and show a mixed CXCR5^+^/CXCR5^-^ phenotype

We previously showed that antigen-specific blood CD4^+^IL-21^+^ICOS^+^ T_H_ cells expand three weeks after influenza vaccination [17]. In the present study, we extended these observations by measuring the frequency of circulating H1N1-specific CD4^+^IL-21^+^ICOS^+^ T_H_ cells as early as 7 days after vaccination. PBMCs were stimulated overnight with A/California/7/2009 (H1N1) subunit antigen, and IL-21-producing cells were identified by flow cytometry (S1C Fig for subsets identification).

**Fig 4:**
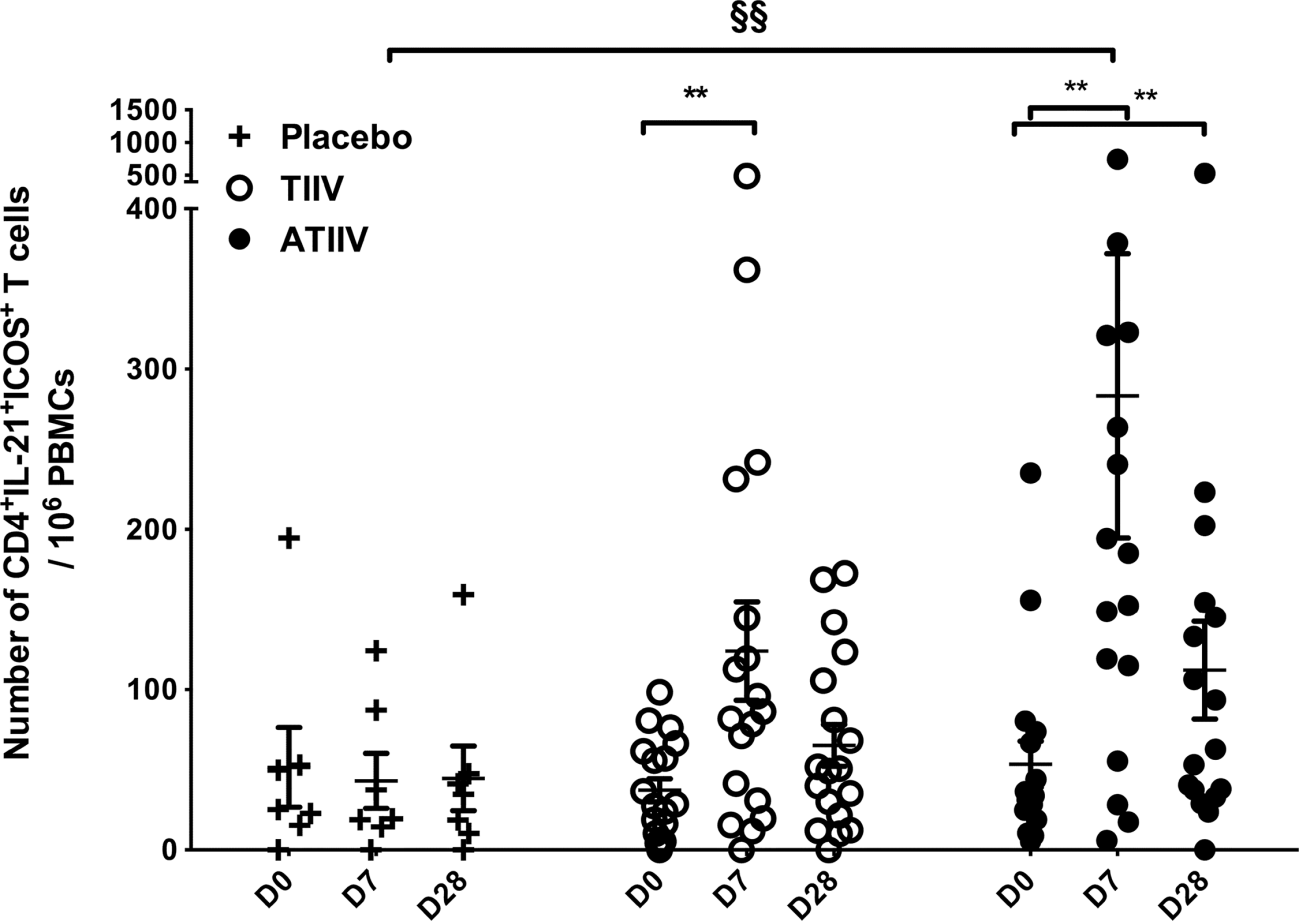
H1N1-specific CD4^+^IL-21^+^ICOS^+^ T_H_ cells expand 7 days after seasonal influenza vaccination. Numbers of CD4^+^IL-21^+^ICOS^+^ T_H_ cells in PBMCs stimulated overnight with A/California/7/2009 (H1N1) antigen. Data show three cohorts: saline placebo (n=7), TIIV (n=18) and ATIIV (n=17) at baseline (D0), day 7 (D7) and day 28 (D28) after a single dose of influenza vaccine. Data are shown for each subject and expressed as number of cells in 10^6^ live CD4^+^ T cells acquired; mean ± SEM is shown. Non-parametric Wilcoxon’s signed rank test was used for statistical analyses: *p < 0.05, **p < 0.01, and ***p < 0.001 compared to day 0; ^§^*p* < 0.05, ^§§^*p* < 0.01 and ^§§§^*p* < 0.001 compared to saline placebo.

H1N1-specific CD4^+^IL-21^+^ICOS^+^ T_H_ cells were already detectable on day 0 (mean values of 51 ± 25, 37 ± 7, and 53 ± 14 cells/10^6^ CD4^+^ T cells in the placebo, TIIV, and ATIIV groups, respectively) suggesting that most of the participants had memory T cells due to prior vaccination or to natural exposure to circulating influenza viruses (Fig 4). Following vaccination with TIIV and ATIIV, the number of H1N1-specific CD4^+^IL-21^+^ICOS^+^ T_H_ cells significantly increased at day 7 compared to day 0, and decreased by day 28 in both groups, although remaining significantly higher than baseline in the ATIIV group (Fig 4). Finally, the increased frequency of CD4^+^IL-21^+^ICOS^+^ T_H_ cells at day 7 in response to ATIIV was significantly higher than in placebo controls, and persisted up to four weeks after vaccination.

**Fig 5:**
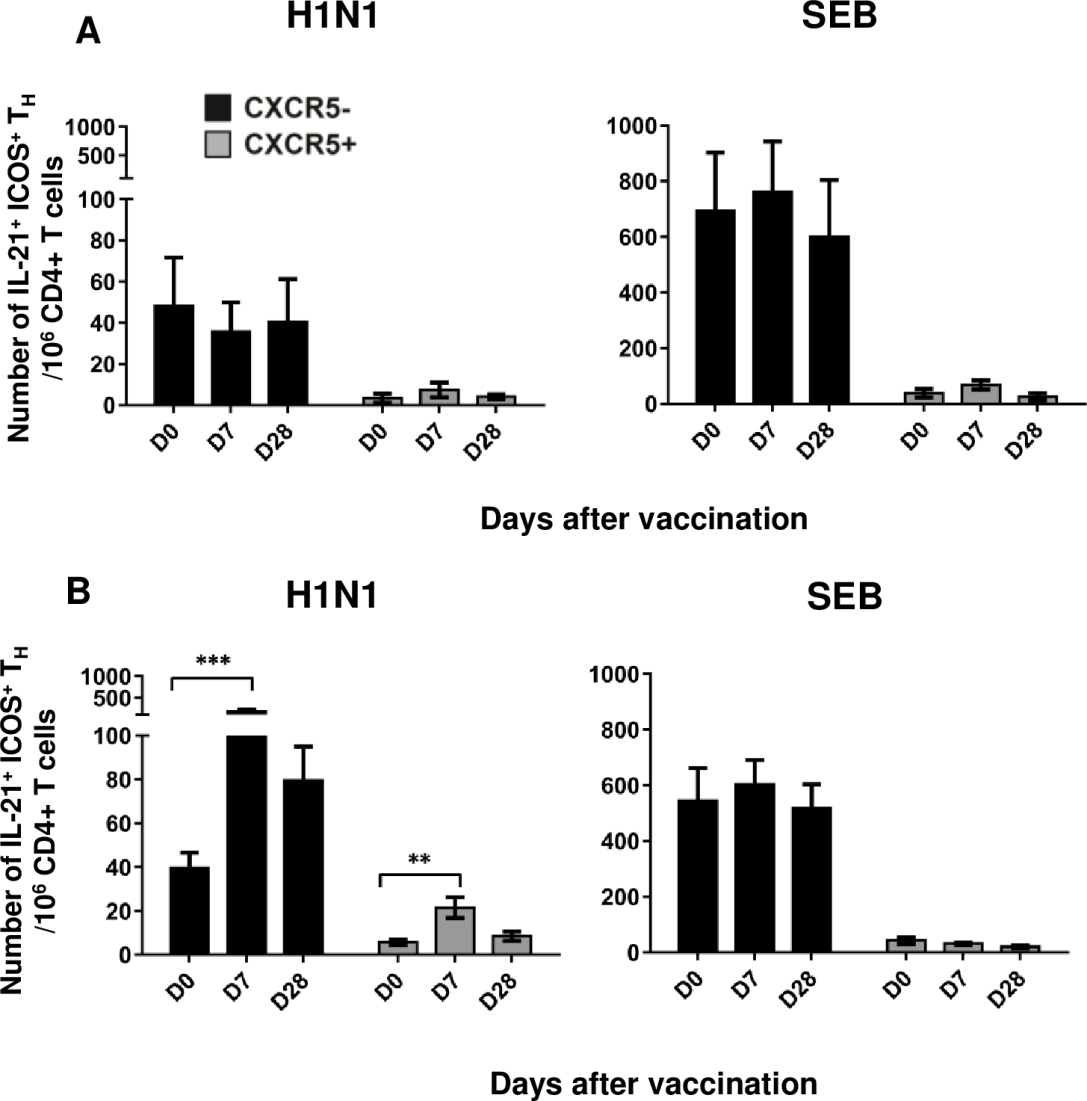
H1N1-specific CD4^+^IL-21^+^ICOS^+^ T_H_ cells subsets expressing or not CXCR5 expand after influenza vaccination. Number of CD4^+^IL-21^+^ICOS^+^ T_H_ cells, showing a CXCR5^+^ (black) or CXCR5^-^ (gray) phenotype, in vaccinated participants after overnight stimulation with A/California/7/2009 (H1N1) antigen or SEB. Data show saline placebo (n=7), and merged TIIV (n=18) and ATIIV (n=17) cohorts at baseline (D0), day 7 (D7) and day 28 (D28) after a single dose of influenza vaccine. Data are expressed as number of cells in 10^6^ live CD4^+^ T cells; mean ± SEM is shown. Non-parametric Wilcoxon’s signed rank test was used for statistical analyses: *p < 0.05, **p < 0.01, and ***p < 0.001 compared to day 0.

We further characterized H1N1-specific CD4^+^IL-21^+^ICOS^+^ T_H_ cells at day 7 based on the level of CXCR5 expression. Due to the intrinsic variability of the single participants and due to the limited number of participants tested, we investigated this parameter by pooling together the results from the TIIV and ATIIV groups. At baseline, H1N1-specific CD4^+^IL-21^+^ICOS^+^ CXCR5^-^ T_H_ cells were more abundant than CXCR5^+^ T_FH_ cells with 40 ± 7 cells/ 10^6^ CD4^+^ T cells and 6 ± 1 cells/ 10^6^ CD4^+^ T cells, respectively (Fig 5), in agreement with our previous results (18). Both subsets expanded at day 7, to 180 ± 44 CD4^+^IL-21^+^ICOS^+^CXCR5^-^ T_H_ cells/ 10^6^ CD4^+^ T cells and 21 ± 5 CD4^+^IL-21^+^ICOS^+^CXCR5^+^ T_FH_ cells/ 10^6^ CD4^+^ T cells, respectively. At day 28, the frequency of H1N1-specific CD4^+^IL-21^+^ICOS^+^CXCR5^+^ T_FH_ cells had returned to baseline levels, while CD4^+^IL-21^+^ICOS^+^CXCR5^-^ T_H_ cells were still high (80 ± 16 cells/ 10^6^ CD4^+^ T cells). In the placebo group, the frequency of H1N1-specific CD4^+^IL-21^+^ICOS^+^ T_H_ cells CXCR5^-^ or CXCR5^+^ did not change over time, suggesting that the expansion of these subsets in TIIV and ATIIV groups was specifically driven by the vaccines.

### Frequencies of T_FH_ cells at day 7 correlate with functional antibody responses after influenza vaccination

We further asked whether the expansion, at day 7, of total CD4^+^ T_FH_1 ICOS^+^ cells or H1N1-specific CD4^+^IL-21^+^ICOS^+^CXCR5^+^ T_FH_ cells correlated with the rise of HI titers measured at day 28 and 168 post-immunization. In contrast to our previous study in which the vaccinees had no appreciable preexposure to avian A/Vietnam/1194/2004 (H5N1) influenza strain [18], most participants in this study had detectable levels of preexsisitng antibodies specific to one or more strains present in the 2012-2013 seasonal influenza vaccine. Baseline HI titers were inversely associated with HI titers measured at day 28 and 168 after vaccination and reached statistical significance for A/California/7/2009 (H1N1) and A/Victoria/361/2011 (H3N2) strains (S4 Fig). Therefore, in order to determine correlates of vaccine immunogenicity that were independent from vaccinees’ baseline preexisting immunity, we defined an alternative endpoint metric: decorrelated hemagglutination inhibition (DHI), which removed any linear correlation between the antibody expansion after vaccination and day 0 HI titers (S5 Fig). Total CD4^+^ T_FH_1 ICOS^+^ cells were correlated to the maximun HI response observed across the three strains represented in the vaccine, while H1N1-specific CD4^+^IL-21^+^ICOS^+^CXCR5^+^ T_FH_ cells were correlated to H1N1-specific HI titers.

**Fig 6:**
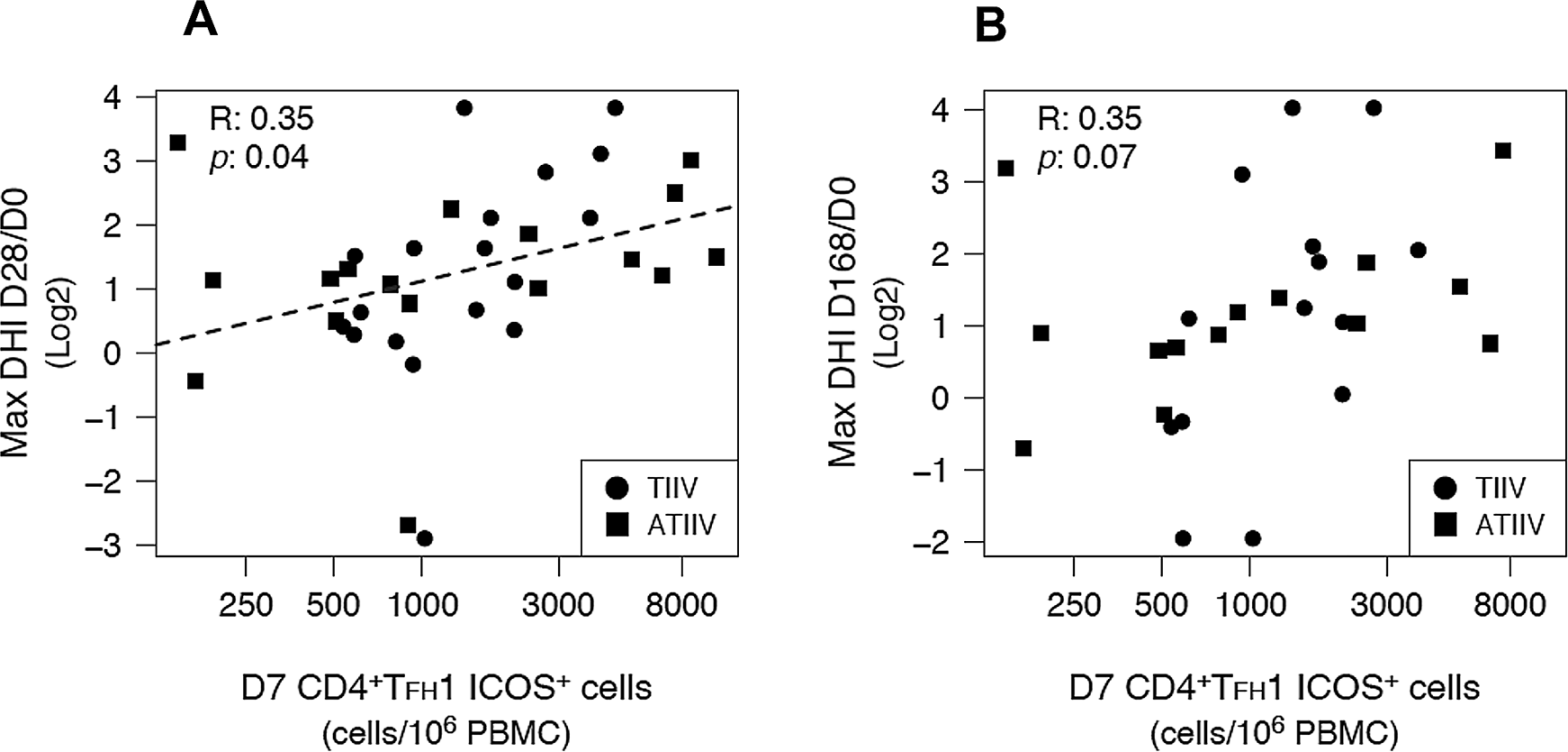
T_FH_1 ICOS^+^ cells predict functional antibody responses. Correlations between the number of total circulating CD4^+^ T_FH_1 ICOS^+^ cells and the maximum DHI responses observed across the three influenza strains represented in the vaccine, measured at (A) day 28 and (B) day 168 after immunizzation. Dashed lines represent the least squares regressions fit to the data. R: Pearson product-moment correlation coefficient. *p*: correlation-associated p value.

**Fig 7:**
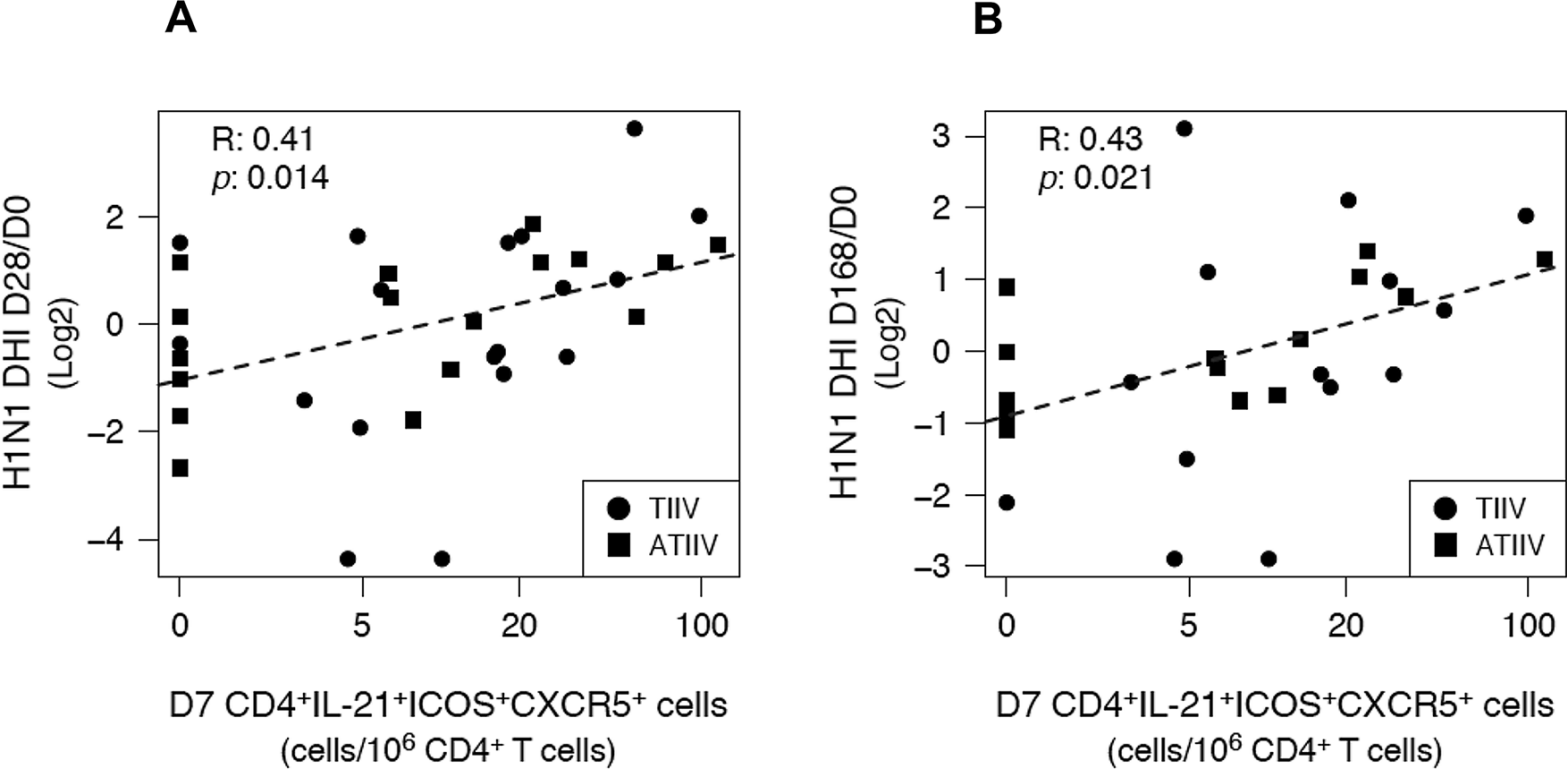
H1N1-specific CD4^+^IL-21^+^ICOS^+^CXCR5^+^ T_FH_ cells predict functional antibody responses. Correlations between the number of H1N1-specific CD4^+^IL-21^+^ICOS^+^CXCR5^+^ T_FH_ cells and H1N1-specific DHI responses measured at (A) day 28 and (B) day 168 after immunizzation. Dashed lines represent the least squares regressions fit to the data. R: Pearson product-moment correlation coefficient. *p*: correlation-associated p value.

The number of CD4^+^ T_FH_1 ICOS^+^ cells measured at day 7 after immunization was weakly correlated with DHI responses at day 28 (R = 0.35; n = 35; *p* = 0.04), but not at day 168 (R = 0.35; n = 28; *p* = 0.07) after vaccination (Table 1 and Fig 6). In contrast, the number of H1N1-specific CD4^+^IL-21^+^ICOS^+^CXCR5^+^ T_FH_ cells, measured at day 7, was significanly correlated with both day 28 (R = 0.41; n = 35; *p* = 0.01) and day 168 (R = 0.43; n = 28; *p* = 0.02) DHI responses (Table 1 and Fig 7). H1N1-specific CD4^+^IL-21^+^ICOS^+^ and CD4^+^IL-21^+^ICOS^+^CXCR5^-^ T_H_ cells were not significantly associated with DHI responses at any time point. Interestingly, plasmablasts did not show any significant association with DHI responses at any time point (Table 1), while the frequency of plasmablasts observed at day 7 post-vaccination was negatively correlated with day 0 HI titers (R = -0.46; n = 31; *p* = 0.01) (S6 Fig), suggesting that plasmablast responses to vaccination were also affected by the preexisting immunity status of the vaccinees.

**Table 1:** Correlation between cell subsets and DHI titers.

^a^Predictor	Pearson R	
Day7	DHI Day28/Day0	DHI Day168/Day0
Plasmablasts	^b^0.20	^b^0.23
CD4^+^T_FH_1 ICOS^+^ cells	^b^0.35*	^b^0.35
H1N1-specific CD4^+^IL-21^+^ICOS^+^ T_H_ cells	^c^0.23	^c^0.30
H1N1-specific CD4^+^IL-21^+^ICOS^+^CXCR5^+^ T_FH_ cells	^c^0.41*	^c^0.43*
H1N1-specific CD4^+^IL-21^+^ICOS^+^CXCR5^-^ T_H_ cells	^c^0.20	^c^0.25

Levels of correlation between cell frequencies, measured at day 7, and DHI responses measured 28 and 168 days after vaccination.

* *p* ≤ 0.05.

^a^ All predictors are expressed as number of cells / 10^6^ PBMCs

^b^ Maximum DHI response observed across A/California/7/2009 (H1N1), A/Victoria/361/2011 (H3N2) and B/Wisconsin/1/2010-like vaccine strains.

^c^ A/California/7/2009 (H1N1)-specific DHI responses

In conclusion, our data suggest that the frequencies of both *ex vivo* T_FH_1 ICOS^+^ cells and H1N1-specific CD4^+^IL-21^+^ICOS^+^CXCR5^+^ T_FH_ cells is associated with vaccine-induced functional antibody responses, independently of the vaccinees’ pre-immune status.

## Discussion

In the present study, we have shown that various subpopulations of T cells sharing phenotypic and functional properties with T_FH_ cells are present in the peripheral blood after vaccination with adjuvanted or non-adjuvanted influenza vaccines. The frequency of these blood T cell subpopulations peaks 7 days post-vaccination, then declines to baseline levels, although some (i.e. H1N1-specific CD4^+^IL-21^+^ICOS^+^CXCR5^-^ T_H_ cells) persist for at least four weeks. Frequencies of some T_FH_ cell subsets, either total or antigen-specific, predicted HI titers, not only four weeks after vaccination, but also six months later.

T_FH_ cells are acquiring an increasing interest because of their ability to provide help to B cells in the GC of secondary lymphoid organs, leading to B-cell differentiation, reshaping of the B-cell repertoire, and Ig affinity maturation [1–5]. The role of T_FH_ cells in the generation of long-lived humoral responses to antigens gives them an ideal profile as potential early biomarkers of effective take of vaccines and possibly of long-lasting protective antibody responses. Peripheral T_FH_ subsets in whole blood or fresh human PBMCs of healthy participants have been identified by *ex vivo* staining following vaccination against influenza, describing memory CD4^+^CXCR5^+^ T_H_ cells as the most abundant population [13, 17, 31, 32]. In this study performed on frozen PBMCs, a peak expansion of CD4^+^CXCR5^+^ T_FH_1 cells expressing ICOS and PD-1 was detected 7 days after TIIV vaccination, while T_FH_2 and T_FH_17 cell subsets were not modulated, confirming previous observations by Bentebibel et al. [17]. Similar responses were also observed after ATIIV vaccination, suggesting that the presence of the adjuvant did not alter the profile of these T_FH_ subsets.

We recently reported that H5N1-specific CD4^+^IL-21^+^ICOS^+^ T_H_ cells expanded 21 days after vaccination, showed a CXCR5^-^ phenotype, and positively correlated with the H5N1-specific antibody response after the second vaccine dose [18]. Here, we extended these observations by showing that the frequency of CD4^+^IL-21^+^ICOS^+^ T_H_ cells, specific for the seasonal influenza A/California/7/2009 (H1N1) antigen, increased seven days after influenza vaccination and decreased by day 28. Both CXCR5^-^ and CXCR5^+^ subsets expanded seven days after influenza vaccination, while at day 28, most CD4^+^IL-21^+^ICOS^+^ T_H_ cells were CXCR5^-^, suggesting that antigen-specific memory T_FH_ cells might be in a resting state in the periphery and transiently express CXCR5 homing receptor upon antigen encounter.

A number of independent studies have reported that preexisting antigen-specific antibodies affect responsiveness to influenza vaccination, with higher baseline levels associated with lower HI responses [33]. Therefore, we defined a new endpoint metric, DHI, that decorralated antibody expansion after vaccination from day 0 HI titers. This new endpoint enabled us to determine cellular correlates of vaccine immunogenicity that were independent from the vaccinees’ preexisting immunity to influenza vaccine strains. With this approach, we showed that frequencies of plasmablasts at day 7 did not correlate with later DHI responses, suggesting that previously described associations between plasmablasts and antibody responses [17] might not be based on a causal effect but rather linked to preexisting immunity and, as such, appear correlated when directly compared. In contrast, T_FH_1 ICOS^+^ cells were shown to have some predictive power for day 28 DHI responses, confirming previous observations [17], but not for day 168 DHI responses. In addition, we also showed that day 7 H1N1-specific CD4^+^IL-21^+^ICOS^+^CXCR5^+^ T_FH_ cell responses were significantly correlated with both early and late H1N1-specific antibody responses, with higher cell frequencies being associated with higher DHI responses. These results highlight the complex interplay between different factors, both subject- and vaccine-related, occurring during an immunological response to vaccination, leading to the production and persistence of functional antibodies.

In the present study, we identified two discrete H1N1-specific T_H_ populations, one being CD4^+^IL-21^+^ICOS^+^CXCR5^+^ and the other lacking the CXCR5 marker. Interestingly, CD4^+^IL-21^+^ICOS^+^CXCR5^+^ T_FH_ cells expanded transiently after vaccination. These cells may represent a transitional state of a subset of cells belonging to the larger CD4^+^IL-21^+^ICOS^+^CXCR5^-^ T_H_ cell population that persisted at higher frequency in the blood for at least 4 weeks, maintained their antigen-specificity as demonstrated by the ability to produce IL-21 and, as shown previously, by their capacity to provide help to B cells. Nonetheless, the possibility that the antigen-specific CD4^+^IL-21^+^ICOS^+^CXCR5^+^ T_FH_ cells identified in our study represent a small subset of T_FH_1 ICOS^+^ cells cannot be excluded. In any case, these findings provide evidence that both T_FH_1 ICOS^+^ and antigen-specific CD4^+^IL-21^+^ICOS^+^CXCR5^+^ T_FH_ cell subsets are associated with functional antibody responses to influenza vaccination. Further experimentation is needed to test whether the early response of these T_FH_ subsets can be used as biomarkers of later functional immune responses for vaccines other than influenza or for vaccines for which participants have no immunological memory.

Taken together, our data give new insights on T_FH_ cell subset responses after influenza vaccination and on their potential involvement in the persistence of protective antibody levels. We envision that applying similar approaches in future clinical trials will provide new insights into the mechanisms underlying the immunological response to vaccination and advance our ability to prospectively evaluate vaccine responsiveness based on early cellular and molecular signatures. This will represent an important advance in the development of novel or improved vaccines.

## Supporting Information

S1 Fig Gating strategy applied to PBMCs. (**A**) Plasmablasts (**B**) *Ex-vivo* T_FH_ subsets. (**C**) H1N1-specific CD4^+^IL-21^+^ICOS^+^ T_H_ cells.
(ZIP)

S2 Fig TIIV and ATIIV vaccination did not change the frequencies of blood CD4^+^ICOS^+^CXCR5^-^ T_H_ and ICOS^-^CXCR5^+^ T_FH_ cells. Data show three cohorts: saline placebo (n=7), TIIV (n=18) and ATIIV (n=17) at day 0, day 7 and day 28 after a single dose of influenza vaccine. Data are shown for each participant and expressed as number of cells in 10^6^ live PBMCs acquired. Non-parametric Wilcoxon’s signed rank test was used for statistical analyses. *p* > 0.05 compared to day 0 and to saline placebo.
(TIF)

S3 Fig TIIV and ATIIV vaccination did not change ICOS expression in blood T_FH_2 and T_FH_17 subsets. TIIV and ATIIV vaccination did not change ICOS expression in blood T_FH_2 and T_FH_17 subsets. Data show three cohorts: saline placebo (n=7), TIIV (n=18) and ATIIV (n=17) at day 0, day 7 and day 28 after a single dose of influenza vaccine. Data are shown for each participant and expressed as number of cells in 10^6^ live PBMCs acquired. Non-parametric Wilcoxon’s signed rank test was used for statistical analyses. *p* > 0.05 compared to day 0 and to saline placebo.
(TIF)

S4 Fig Correlation between HI titers fold-increase and baseline HI titers. HI titers were determined for A/California/7/2009 (H1N1), A/Victoria/361/2011 (H3N2) and B/Wisconsin/1/2010-like vaccine strains.
(TIF)

S5 Fig Correlation between DHI responses and baseline HI titers. HI titers were determined for A/California/7/2009 (H1N1), A/Victoria/361/2011 (H3N2) and B/Wisconsin/1/2010-like vaccine strains.
(TIF)

S6 Fig Correlation between day 7 plasmablasts frequency and baseline HI titers. Baseline HI titers refer to the maximun value observed across A/California/7/2009 (H1N1), A/Victoria/361/2011 (H3N2) and B/Wisconsin/1/2010-like vaccine strains. Dashed lines represent the least squares regressions fit to the data. R: Pearson product-moment correlation coefficient. *p*: correlation associated p value.
(TIF)

S1 Table Rates of protection against all vaccine strains. (DOCX)

S2 Table HI Titers. (PDF)

S3 Table Frequency of Plasmablasts. (PDF)

S4 Table Frequency of Antigen-specific T cells. (PDF)

S5 Table Frequency of *Ex vivo* CD4 T cells. (PDF)

S1 Text Clinical Trial Protocol CRC305C. (PDF)

S2 Text CONSORT Checklist. (DOCX)
