# Supplementary figures and images for: Early Rise of Blood T Follicular Helper Cell Subsets and Baseline Immunity as Predictors of Persisting Late Functional Antibody Responses to Vaccination in Humans
Source: PLoS One. 2016 Jun 23;11(6):e0157066. doi: 10.1371/journal.pone.0157066 (PMC4918887; doi:10.1371/journal.pone.0157066)

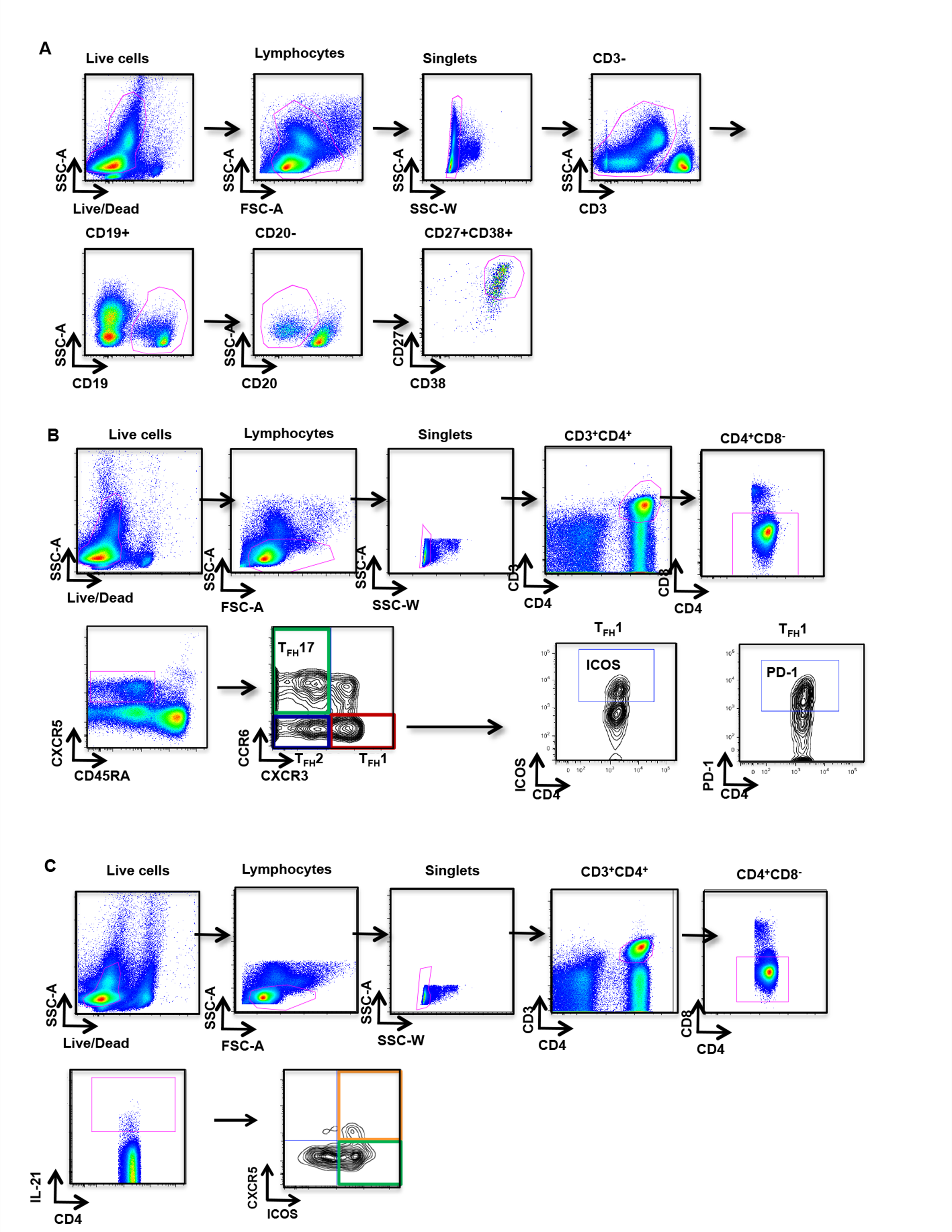

Supplement: S1 Fig — (A) Plasmablasts (B) Ex-vivo TFH subsets. (C) H1N1-specific CD4+IL-21+ICOS+ TH cells. (ZIP) [file pone.0157066.s001.zip › Figure S1.tif]

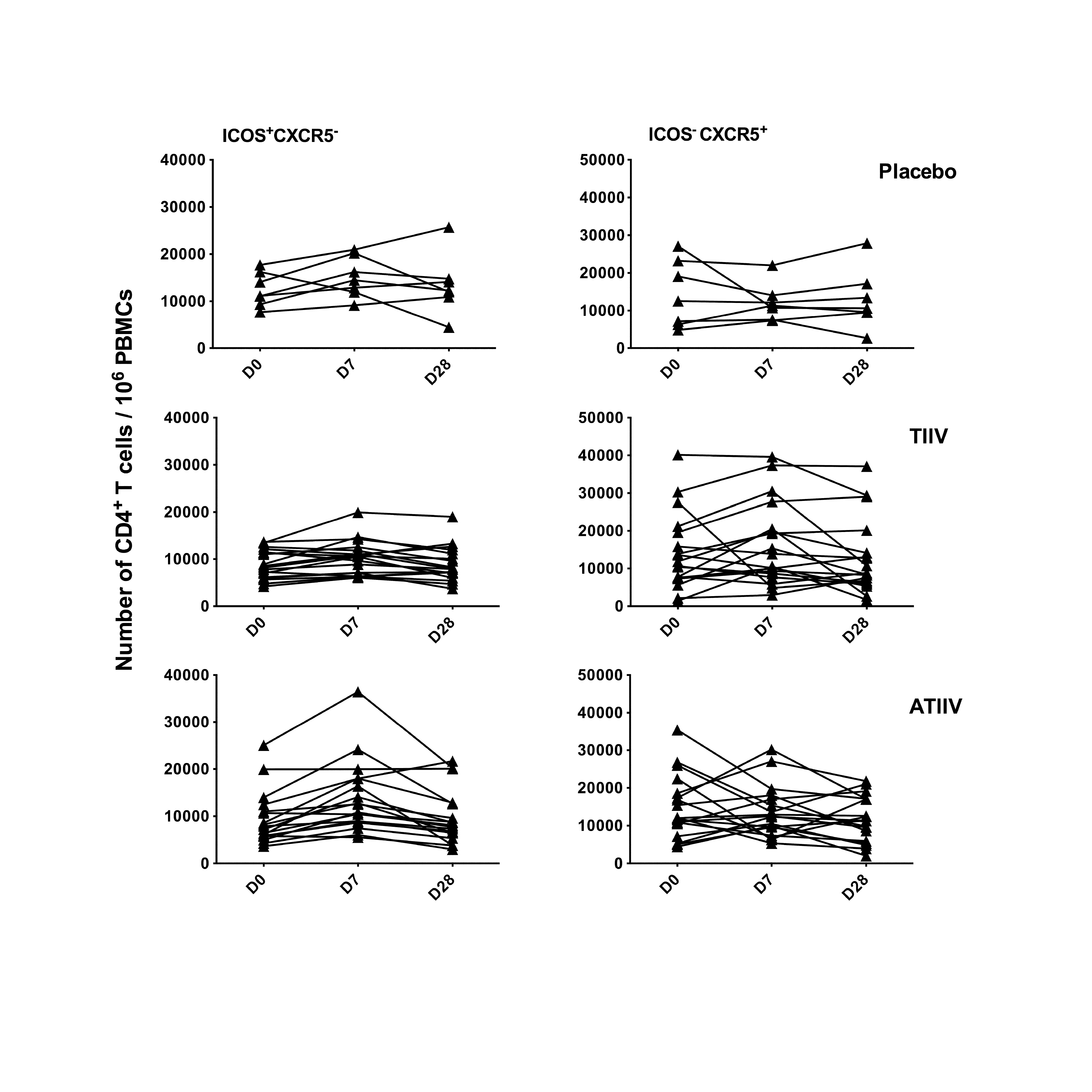

Supplement: S2 Fig — Data show three cohorts: saline placebo (n=7), TIIV (n=18) and ATIIV (n=17) at day 0, day 7 and day 28 after a single dose of influenza vaccine. Data are shown for each participant and expressed as number of cells in 106 live PBMCs acquired. Non-parametric Wilcoxon’s signed rank test was used for statistical analyses. p > 0.05 compared to day 0 and to saline placebo. (TIF) [file pone.0157066.s002.tif]

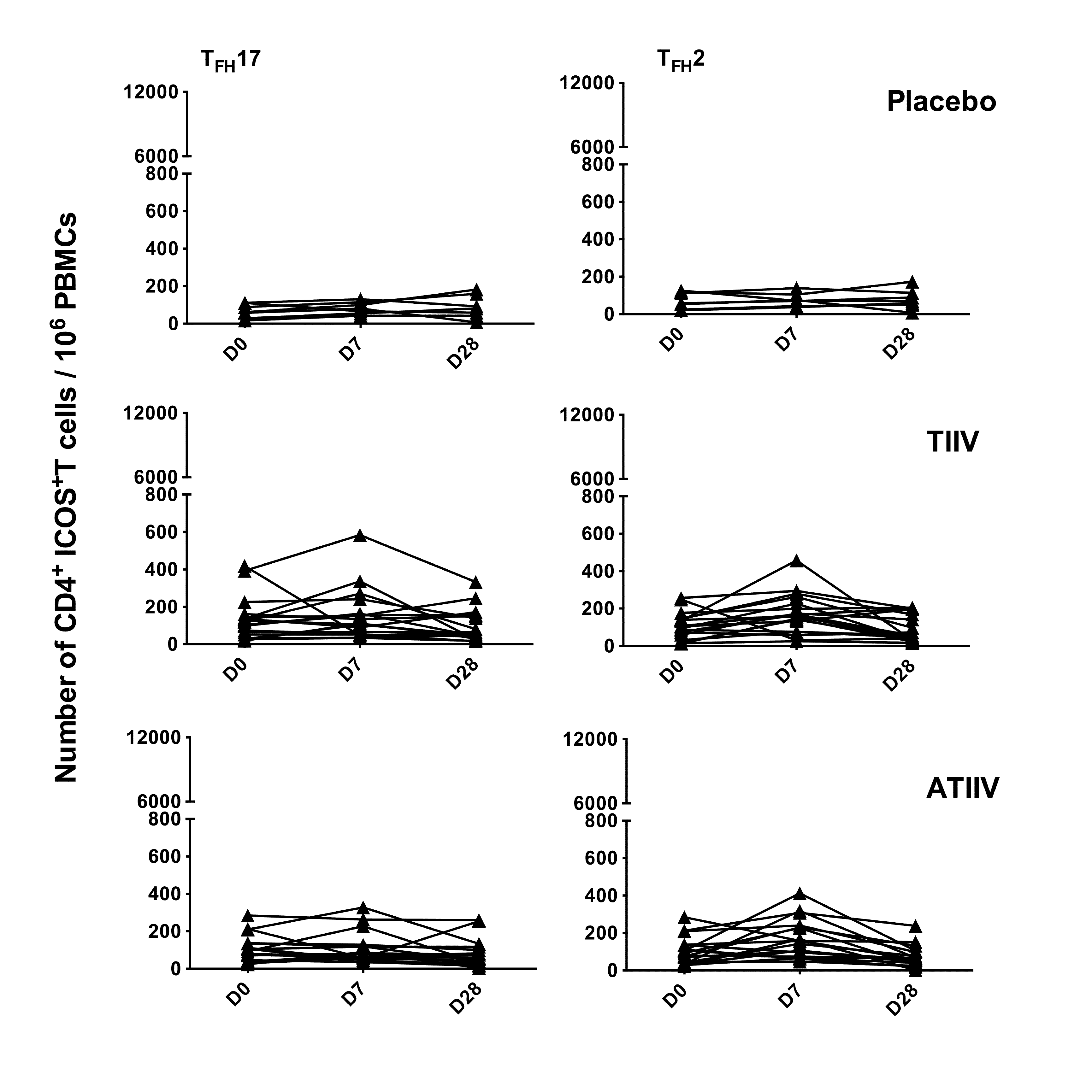

Supplement: S3 Fig — TIIV and ATIIV vaccination did not change ICOS expression in blood TFH2 and TFH17 subsets. Data show three cohorts: saline placebo (n=7), TIIV (n=18) and ATIIV (n=17) at day 0, day 7 and day 28 after a single dose of influenza vaccine. Data are shown for each participant and expressed as number of cells in 106 live PBMCs acquired. Non-parametric Wilcoxon’s signed rank test was used for statistical analyses. p > 0.05 compared to day 0 and to saline placebo. (TIF) [file pone.0157066.s003.tif]

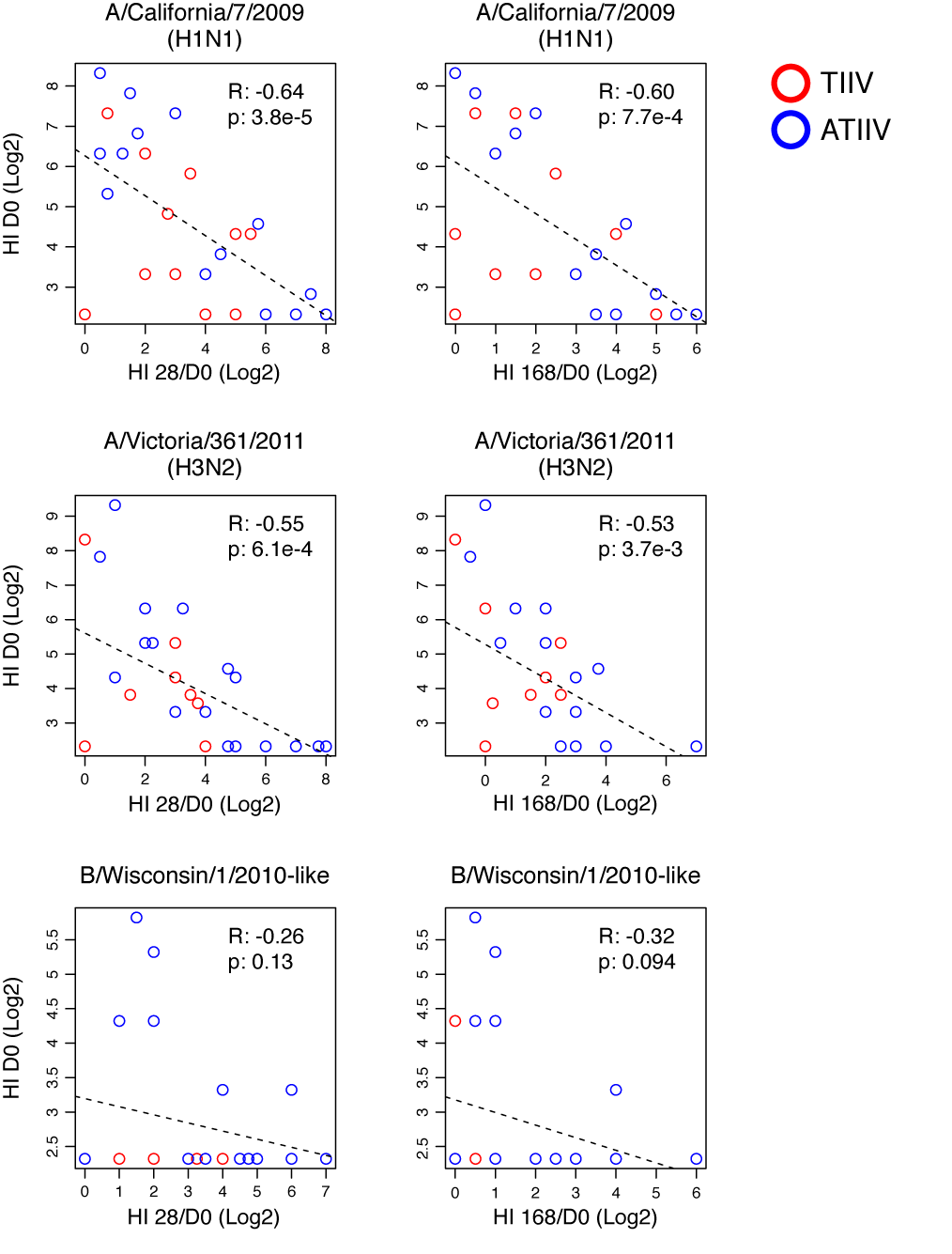

Supplement: S4 Fig — HI titers were determined for A/California/7/2009 (H1N1), A/Victoria/361/2011 (H3N2) and B/Wisconsin/1/2010-like vaccine strains. (TIF) [file pone.0157066.s004.tif]

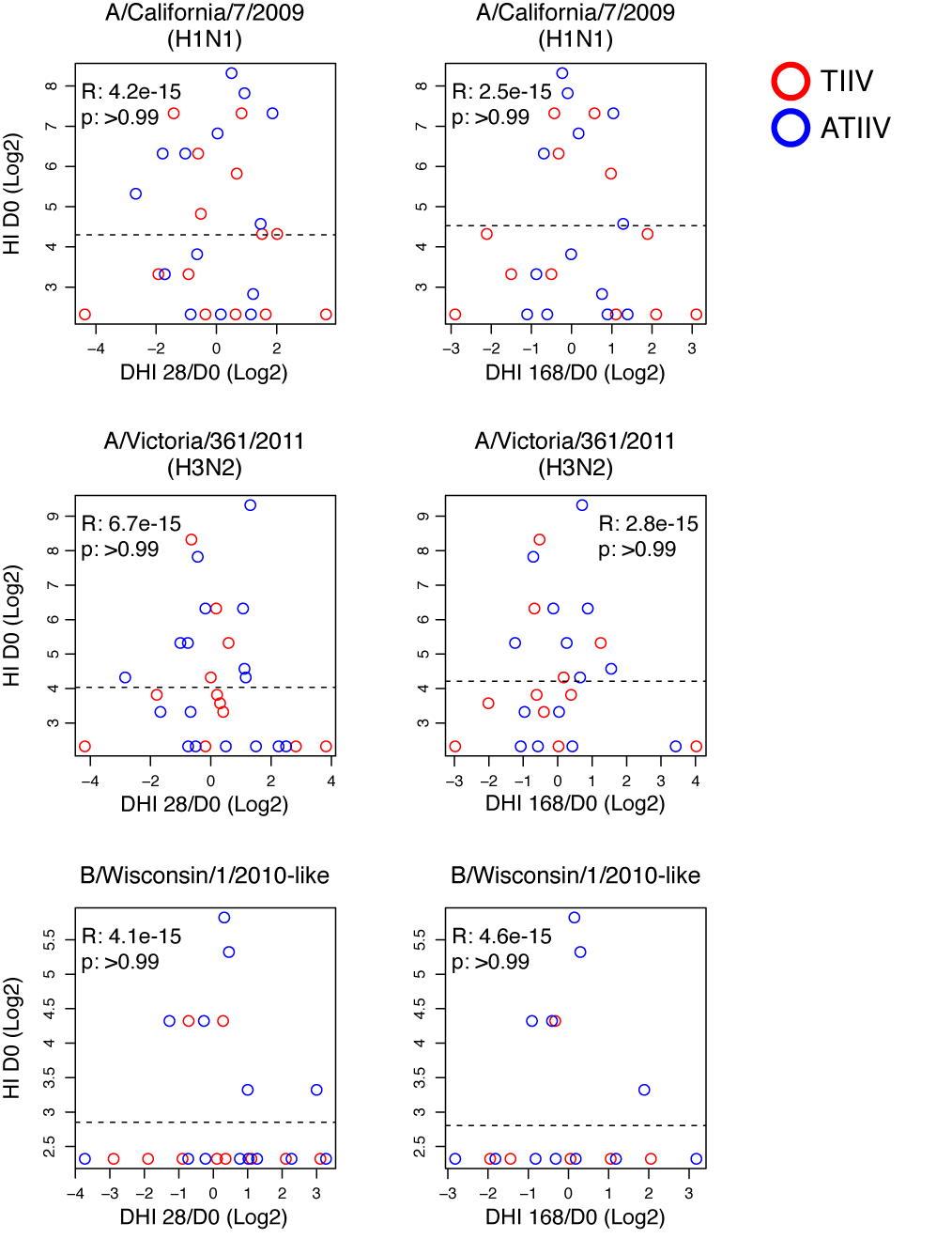

Supplement: S5 Fig — HI titers were determined for A/California/7/2009 (H1N1), A/Victoria/361/2011 (H3N2) and B/Wisconsin/1/2010-like vaccine strains. (TIF) [file pone.0157066.s005.tif]

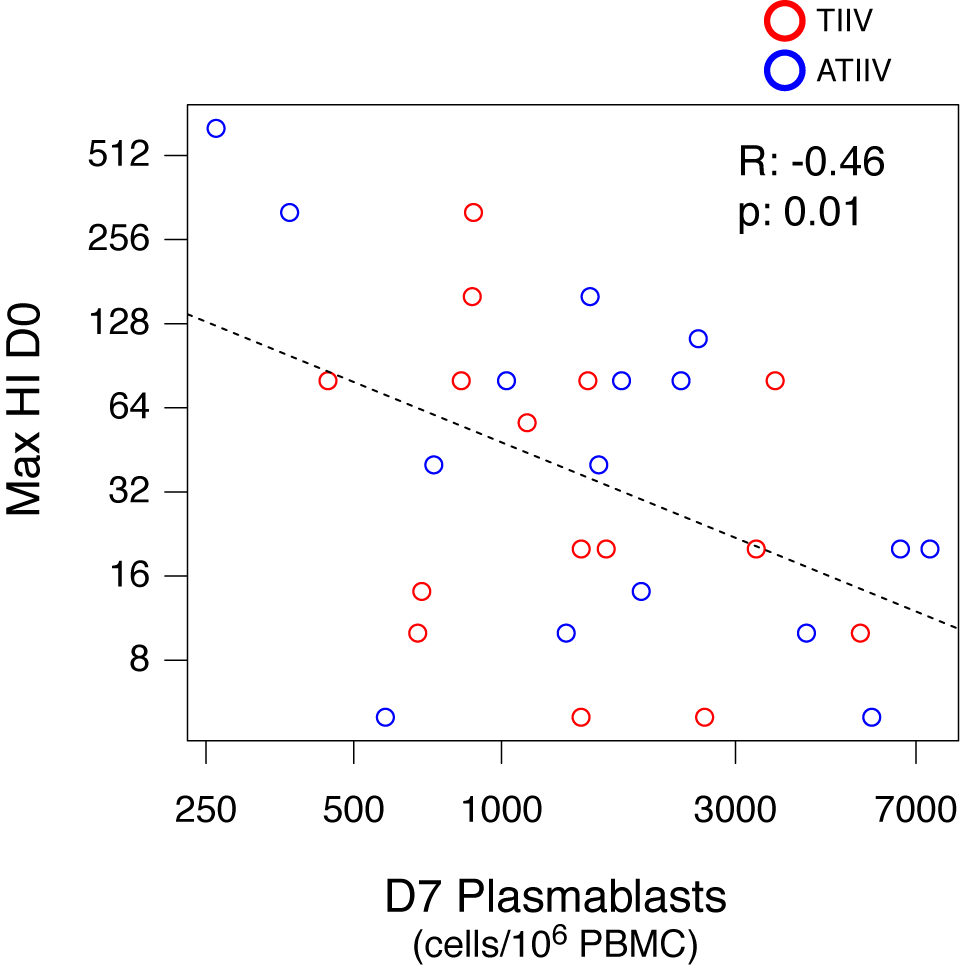

Supplement: S6 Fig — Baseline HI titers refer to the maximun value observed across A/California/7/2009 (H1N1), A/Victoria/361/2011 (H3N2) and B/Wisconsin/1/2010-like vaccine strains. Dashed lines represent the least squares regressions fit to the data. R: Pearson product-moment correlation coefficient. p: correlation associated p value. (TIF) [file pone.0157066.s006.tif]
