# Supplementary material for: Early Rise of Blood T Follicular Helper Cell Subsets and Baseline Immunity as Predictors of Persisting Late Functional Antibody Responses to Vaccination in Humans
Source: PLoS One. 2016 Jun 23;11(6):e0157066. doi: 10.1371/journal.pone.0157066 (PMC4918887; doi:10.1371/journal.pone.0157066)
Supplement: S1 Table — (DOCX) [file pone.0157066.s007.docx]

**Table S1. Rates of seroprotection against all vaccine strains**

| **Seroprotection Rates (HI ≥ 1:40)** | | | | |
| --- | --- | --- | --- | --- |
|  | | **Placebo** | **TIIV** | **ATIIV** |
| **A/California/7/2009 (H1N1)** | **D0** | 12.5% | 29% | 40% |
|  | **D7** | 25% | 86% | 95% |
|  | **D28** | 25% | 90% | 100% |
|  | **D168** | 33% | 73% | 100% |
| **A/Victoria/361/2011 (H3N2)** | **D0** | 0 | 29% | 40% |
|  | **D7** | 0 | 81% | 100% |
|  | **D28** | 0 | 86% | 100% |
|  | **D168** | 0 | 73% | 94% |
| **B/Wisconsin/1/**  **2010-like** | **D0** | 0 | 0 | 10% |
|  | **D7** | 0 | 43% | 80% |
|  | **D28** | 0 | 52% | 90% |
|  | **D168** | 0 | 40% | 56% |
