# Supplementary material for: Early Rise of Blood T Follicular Helper Cell Subsets and Baseline Immunity as Predictors of Persisting Late Functional Antibody Responses to Vaccination in Humans
Source: PLoS One. 2016 Jun 23;11(6):e0157066. doi: 10.1371/journal.pone.0157066 (PMC4918887; doi:10.1371/journal.pone.0157066)
Supplement: S2 Table — (PDF) [file pone.0157066.s008.pdf]

S1 Table HI titers

	Antigen: A/California/7/2009				Antigen: A/Victoria/361/2011			
	Geometric Mean HAI titre				Geometric Mean HAI titre			
Sample ID	DAY0	DAY7	DAY21	DAY168	DAY0	DAY7	DAY21	DAY168
9001	5	640	320	N/A	5	640	320	N/A
9004	20	320	640	N/A	10	160	160	N/A
9010	5	320	320	80	5	160	160	40
9011	5	5	5	5	5	5	5	5
9012	20	80	135	40	80	160	320	160
9014	5	761	1280	640	160	320	640	453
9015	40	80	80	40	40	80	80	40
9017	20	640	905	320	20	80	160	80
9018	10	80	40	20	80	320	320	80
9021	5	80	320	160	14	80	160	80
9022	5	40	40	N/A	5	5	5	N/A
9024	5	5	5	N/A	5	5	5	N/A
9026	320	453	453	320	40	160	160	57
9027	80	320	320	160	5	80	80	40
9028	10	80	80	40	5	5	5	5
9031	5	1280	1280	320	5	1280	1076	80
9033	80	320	320	160	14	40	40	40
9035	5	5	5	5	10	80	160	40
9036	5	5	5	N/A	5	5	5	N/A
9038	5	5	5	N/A	5	5	5	N/A
9041	7	1280	1280	226	10	80	80	40
9043	5	640	640	N/A	5	320	320	N/A
9044	5	1280	1280	N/A	5	160	640	N/A
9045	160	1280	1280	453	5	640	640	640
9046	10	80	160	80	20	538	640	160
9050	5	1280	1280	226	80	226	320	160
9051	5	5	80	N/A	5	5	80	N/A
9055	5	5	5	5	5	5	5	5
9056	80	160	190	160	226	320	320	160
9057	20	1280	640	20	12	320	160	14
9060	20	135	640	N/A	20	40	226	N/A
9061	28	95	190	N/A	80	135	320	N/A
9062	160	320	269	226	5	761	1280	640
9063	160	1280	1280	640	40	320	190	160
9065	57	320	640	320	40	160	320	226
9066	226	640	640	320	640	640	1280	640
9068	113	640	381	320	5	381	1280	640
9069	5	5	640	57	80	80	761	320
9071	5	113	320	320	320	320	320	160
9072	640	905	761	N/A	160	538	640	N/A
9073	24	1280	1280	453	24	640	640	320
9074	5	5	5	5	5	5	5	5
9078	14	226	320	160	5	57	135	28

9080	160	160	160	160	5	5	5	5
9081	5	5	5	N/A	5	5	5	N/A
9082	80	80	113	160	10	40	160	80
9083	5	1280	1280	N/A	5	640	1280	N/A
9088	40	67	67	N/A	20	40	40	N/A
9091	5	80	160	80	5	5	5	5

Antigen: B/HubeiWujiagang/158/2009

Geometric Mean HAI titre			
DAY0	DAY7	DAY21	DAY168
7	80	57	N/A
5	453	320	N/A
5	160	113	80
5	5	5	5
5	5	5	5
5	7	67	5
5	5	40	20
5	40	160	40
20	80	80	20
5	5	5	5
5	5	5	N/A
5	5	5	N/A
20	40	40	40
5	80	48	20
5	160	160	80
5	226	160	80
5	40	80	40
5	5	20	7
5	5	5	N/A
5	5	5	N/A
5	40	5	5
10	320	640	N/A
20	80	80	N/A
5	160	160	80
5	40	57	28
5	40	40	10
20	34	40	N/A
5	5	5	5
20	40	40	28
5	20	10	5
5	5	5	N/A
5	5	40	N/A
5	226	160	80
5	160	160	40
5	5	5	5
57	80	160	80
5	640	320	80
5	5	135	20
5	80	80	40
5	5	5	N/A
40	160	160	80
5	5	5	5
5	320	640	320

5	5	5	5
5	5	5	N/A
10	40	160	160
5	5	5	N/A
5	10	5	N/A
5	5	5	5
