# Supplementary material for: Early Rise of Blood T Follicular Helper Cell Subsets and Baseline Immunity as Predictors of Persisting Late Functional Antibody Responses to Vaccination in Humans
Source: PLoS One. 2016 Jun 23;11(6):e0157066. doi: 10.1371/journal.pone.0157066 (PMC4918887; doi:10.1371/journal.pone.0157066)
Supplement: S3 Table — (PDF) [file pone.0157066.s009.pdf]

S3 Table Frequency of Plasmablasts

			X10^6 PBMCs
SAMPLE I	PATIENT ID		
9004	day 0	TIIV	421
9004	day 7	TIIV	1635
9004	day 28	TIIV	96
9017	day 0	TIIV	997
9017	day 7	TIIV	3306
9017	day 28	TIIV	446
9018	day 0	TIIV	266
9018	day 7	TIIV	443
9018	day 28	TIIV	360
9021	day 0	TIIV	669
9021	day 7	TIIV	688
9021	day 28	TIIV	364
9027	day 0	TIIV	438
9027	day 7	TIIV	828
9027	day 28	TIIV	386
9028	day 0	TIIV	928
9028	day 7	TIIV	5390
9028	day 28	TIIV	1475
9033	day 0	TIIV	43
9033	day 7	TIIV	1500
9033	day 28	TIIV	73
9035	day 0	TIIV	428
9035	day 7	TIIV	675
9035	day 28	TIIV	277
9045	day 0	TIIV	N/A
9045	day 7	TIIV	N/A
9045	day 28	TIIV	N/A
9051	day 0	TIIV	N/A
9051	day 7	TIIV	N/A
9051	day 28	TIIV	N/A
9055	day 0	TIIV	N/A
9055	day 7	TIIV	N/A
9055	day 28	TIIV	N/A
9057	day 0	TIIV	255
9057	day 7	TIIV	1454
9057	day 28	TIIV	241
9061	day 0	TIIV	1748
9061	day 7	TIIV	3613
9061	day 28	TIIV	734
9062	day 0	TIIV	328
9062	day 7	TIIV	872

9062 day 28	TIIV	238
9065 day 0	TIIV	188
9065 day 7	TIIV	1128
9065 day 28	TIIV	131
9071 day 0	TIIV	204
9071 day 7	TIIV	877
9071 day 28	TIIV	212
9083 day 0	TIIV	289
9083 day 7	TIIV	1453
9083 day 28	TIIV	894
9091 day 0	TIIV	1143
9091 day 7	TIIV	2595
9091 day 28	TIIV	1342
9010 day 0	ATIIV	1418
9010 day 7	ATIIV	5686
9010 day 28	ATIIV	1223
9026 day 0	ATIIV	209
9026 day 7	ATIIV	370
9026 day 28	ATIIV	248
9031 day 0	ATIIV	140
9031 day 7	ATIIV	580
9031 day 28	ATIIV	43
9041 day 0	ATIIV	68
9041 day 7	ATIIV	1355
9041 day 28	ATIIV	102
9043 day 0	ATIIV	0
9043 day 7	ATIIV	4185
9043 day 28	ATIIV	165
9044 day 0	ATIIV	338
9044 day 7	ATIIV	7472
9044 day 28	ATIIV	342
9046 day 0	ATIIV	633
9046 day 7	ATIIV	6505
9046 day 28	ATIIV	447
9050 day 0	ATIIV	667
9050 day 7	ATIIV	1024
9050 day 28	ATIIV	N/A
9056 day 0	ATIIV	660
9056 day 7	ATIIV	N/A
9056 day 28	ATIIV	117
9063 day 0	ATIIV	340
9063 day 7	ATIIV	1516
9063 day 28	ATIIV	253
9066 day 0	ATIIV	107

9066	day 7	ATIIV	262
9066	day 28	ATIIV	81
9068	day 0	ATIIV	370
9068	day 7	ATIIV	2519
9068	day 28	ATIIV	643
9069	day 0	ATIIV	320
9069	day 7	ATIIV	2323
9069	day 28	ATIIV	274
9073	day 0	ATIIV	122
9073	day 7	ATIIV	1579
9073	day 28	ATIIV	204
9078	day 0	ATIIV	255
9078	day 7	ATIIV	1927
9078	day 28	ATIIV	178
9082	day 0	ATIIV	266
9082	day 7	ATIIV	1757
9082	day 28	ATIIV	362
9088	day 0	ATIIV	1756
9088	day 7	ATIIV	728
9088	day 28	ATIIV	858
9022	day 0	PLACEBO	319
9022	day 7	PLACEBO	434
9022	day 28	PLACEBO	775
9024	day 0	PLACEBO	414
9024	day 7	PLACEBO	650
9024	day 28	PLACEBO	447
9036	day 0	PLACEBO	822
9036	day 7	PLACEBO	1750
9036	day 28	PLACEBO	670
9038	day 0	PLACEBO	381
9038	day 7	PLACEBO	1084
9038	day 28	PLACEBO	256
9074	day 0	PLACEBO	449
9074	day 7	PLACEBO	352
9074	day 28	PLACEBO	477
9080	day 0	PLACEBO	174
9080	day 7	PLACEBO	214
9080	day 28	PLACEBO	259
9081	day 0	PLACEBO	237
9081	day 7	PLACEBO	245
9081	day 28	PLACEBO	719

*Number of CD20-/CD27++CD38++
