# Supplementary material for: Early Rise of Blood T Follicular Helper Cell Subsets and Baseline Immunity as Predictors of Persisting Late Functional Antibody Responses to Vaccination in Humans
Source: PLoS One. 2016 Jun 23;11(6):e0157066. doi: 10.1371/journal.pone.0157066 (PMC4918887; doi:10.1371/journal.pone.0157066)
Supplement: S4 Table — (PDF) [file pone.0157066.s010.pdf]

S4 Table Frequency of Antigen-specific T cells

SAMPLE ID	PATIENT ID	Time point	Vaccine	CD4+ICOS+CXCR5-IL21+	CD4+ICOS+CXCR5+IL21+	CD4+ICOS+IL21+
9017	medium	day 0	TIIV	0.00	0.00	0.00
9017	H1N1	day 0	TIIV	91.86	6.54	98.40
9017	SEB	day 0	TIIV	699.98	0.00	699.98
9017	medium	day 7	TIIV	0.00	0.00	0.00
9017	H1N1	day 7	TIIV	391.91	97.84	489.75
9017	SEB	day 7	TIIV	967.33	0.00	967.33
9017	medium	day 28	TIIV	0.00	0.00	0.00
9017	H1N1	day 28	TIIV	85.30	38.34	123.64
9017	SEB	day 28	TIIV	831.00	0.00	831.00
9018	medium	day 0	TIIV	0.00	0.00	0.00
9018	H1N1	day 0	TIIV	14.22	4.61	18.83
9018	SEB	day 0	TIIV	204.58	61.12	265.70
9018	medium	day 7	TIIV	0.00	0.00	0.00
9018	H1N1	day 7	TIIV	14.82	4.88	19.70
9018	SEB	day 7	TIIV	69.70	46.50	116.20
9018	medium	day 28	TIIV	0.00	0.00	0.00
9018	H1N1	day 28	TIIV	6.34	6.07	12.41
9018	SEB	day 28	TIIV	6.90	0.00	6.90
9021	medium	day 0	TIIV	0.00	0.00	0.00
9021	H1N1	day 0	TIIV	44.83	12.21	57.04
9021	SEB	day 0	TIIV	730.83	41.65	772.48
9021	medium	day 7	TIIV	0.00	0.00	0.00
9021	H1N1	day 7	TIIV	61.43	20.43	81.86
9021	SEB	day 7	TIIV	718.63	101.40	820.03
9021	medium	day 28	TIIV	0.00	0.00	0.00
9021	H1N1	day 28	TIIV	163.57	5.15	168.72
9021	SEB	day 28	TIIV	950.86	38.40	989.26
9027	medium	day 0	TIIV	0.00	0.00	0.00
9027	H1N1	day 0	TIIV	80.72	0.01	80.73
9027	SEB	day 0	TIIV	3667.74	25.80	3693.54
9027	medium	day 7	TIIV	0.00	0.00	0.00
9027	H1N1	day 7	TIIV	55.78	30.44	86.22
9027	SEB	day 7	TIIV	2588.90	18.90	2607.80
9027	medium	day 28	TIIV	0.00	0.00	0.00
9027	H1N1	day 28	TIIV	136.10	36.59	172.69
9027	SEB	day 28	TIIV	2103.03	13.69	2116.72
9028	medium	day 0	TIIV	0.00	0.00	0.00
9028	H1N1	day 0	TIIV	46.20	9.49	55.69
9028	SEB	day 0	TIIV	1281.01	84.91	1365.92
9028	medium	day 7	TIIV	0.00	0.00	0.00
9028	H1N1	day 7	TIIV	102.10	17.40	119.50
9028	SEB	day 7	TIIV	1454.40	45.90	1500.30
9028	medium	day 28	TIIV	0.00	0.00	0.00
9028	H1N1	day 28	TIIV	93.53	48.64	142.17
9028	SEB	day 28	TIIV	1115.71	119.46	1235.17
9033	medium	day 0	TIIV	0.00	0.00	0.00
9033	H1N1	day 0	TIIV	48.43	17.99	66.42
9033	SEB	day 0	TIIV	382.21	12.50	394.71
9033	medium	day 7	TIIV	0.00	0.00	0.00
9033	H1N1	day 7	TIIV	62.47	15.99	78.46
9033	SEB	day 7	TIIV	309.56	20.13	329.69
9033	medium	day 28	TIIV	0.00	0.00	0.00
9033	H1N1	day 28	TIIV	49.25	0.00	49.25

9033	SEB	day 28	TIIV	196.80	10.40	207.20
9035	medium	day 0	TIIV	0.00	0.00	0.00
9035	H1N1	day 0	TIIV	50.42	25.86	76.28
9035	SEB	day 0	TIIV	402.30	65.48	467.78
9035	medium	day 7	TIIV	0.00	0.00	0.00
9035	H1N1	day 7	TIIV	221.50	10.10	231.60
9035	SEB	day 7	TIIV	292.60	24.20	316.80
9035	medium	day 28	TIIV	0.00	0.00	0.00
9035	H1N1	day 28	TIIV	83.80	21.80	105.60
9035	SEB	day 28	TIIV	168.60	26.00	194.60
9045	medium	day 0	TIIV	0.00	0.00	0.00
9045	H1N1	day 0	TIIV	36.50	0.00	36.50
9045	SEB	day 0	TIIV	924.00	42.00	966.00
9045	medium	day 7	TIIV	0.00	0.00	0.00
9045	H1N1	day 7	TIIV	23.70	47.50	71.20
9045	SEB	day 7	TIIV	564.80	62.80	627.60
9045	medium	day 28	TIIV	0.00	0.00	0.00
9045	H1N1	day 28	TIIV	35.32	0.00	35.32
9045	SEB	day 28	TIIV	647.30	92.30	739.60
9051	medium	day 0	TIIV	0.00	0.00	0.00
9051	H1N1	day 0	TIIV	23.75	4.79	28.54
9051	SEB	day 0	TIIV	95.50	382.00	477.50
9051	medium	day 7	TIIV	0.00	0.00	0.00
9051	H1N1	day 7	TIIV	15.46	0.00	15.46
9051	SEB	day 7	TIIV	344.50	71.70	416.20
9051	medium	day 28	TIIV	0.00	0.00	0.00
9051	H1N1	day 28	TIIV	46.02	5.76	51.78
9051	SEB	day 28	TIIV	425.92	64.62	490.54
9055	medium	day 0	TIIV	0.00	0.00	0.00
9055	H1N1	day 0	TIIV	16.37	0.00	16.37
9055	SEB	day 0	TIIV	251.51	24.52	276.03
9055	medium	day 7	TIIV	0.00	0.00	0.00
9055	H1N1	day 7	TIIV	26.42	4.41	30.83
9055	SEB	day 7	TIIV	331.00	45.36	376.36
9055	medium	day 28	TIIV	0.00	0.00	0.00
9055	H1N1	day 28	TIIV	5.98	5.98	11.96
9055	SEB	day 28	TIIV	190.19	11.18	201.37
9057	medium	day 0	TIIV	0.00	0.00	0.00
9057	H1N1	day 0	TIIV	0.00	0.00	0.00
9057	SEB	day 0	TIIV	203.10	13.50	216.60
9057	medium	day 7	TIIV	0.00	0.00	0.00
9057	H1N1	day 7	TIIV	0.00	0.00	0.00
9057	SEB	day 7	TIIV	158.40	22.60	181.00
9057	medium	day 28	TIIV	0.00	0.00	0.00
9057	H1N1	day 28	TIIV	10.10	0.00	10.10
9057	SEB	day 28	TIIV	103.92	0.00	103.92
9061	medium	day 0	TIIV	0.00	0.00	0.00
9061	H1N1	day 0	TIIV	27.38	0.00	27.38
9061	SEB	day 0	TIIV	279.80	0.00	279.80
9061	medium	day 7	TIIV	0.00	0.00	0.00
9061	H1N1	day 7	TIIV	225.50	16.50	242.00
9061	SEB	day 7	TIIV	545.90	47.70	593.60
9061	medium	day 28	TIIV	0.00	0.00	0.00
9061	H1N1	day 28	TIIV	42.28	8.46	50.74
9061	SEB	day 28	TIIV	306.00	54.00	360.00
9062	medium	day 0	TIIV	0.00	0.00	0.00

9062	H1N1	day 0	TIIV	0.00	3.14	3.14
9062	SEB	day 0	TIIV	180.44	11.52	191.96
9062	medium	day 7	TIIV	0.00	0.00	0.00
9062	H1N1	day 7	TIIV	38.41	2.95	41.36
9062	SEB	day 7	TIIV	101.80	0.00	101.80
9062	medium	day 28	TIIV	0.00	0.00	0.00
9062	H1N1	day 28	TIIV	21.43	0.00	21.43
9062	SEB	day 28	TIIV	0.00	0.00	0.00
9065	medium	day 0	TIIV	0.00	0.00	0.00
9065	H1N1	day 0	TIIV	10.38	0.00	10.38
9065	SEB	day 0	TIIV	0.00	0.00	0.00
9065	medium	day 7	TIIV	0.00	0.00	0.00
9065	H1N1	day 7	TIIV	83.30	29.40	112.70
9065	SEB	day 7	TIIV	418.00	0.00	418.00
9065	medium	day 28	TIIV	0.00	0.00	0.00
9065	H1N1	day 28	TIIV	39.89	0.00	39.89
9065	SEB	day 28	TIIV	396.86	17.83	414.69
9071	medium	day 0	TIIV	0.00	0.00	0.00
9071	H1N1	day 0	TIIV	5.25	0.00	5.25
9071	SEB	day 0	TIIV	250.63	4.65	255.28
9071	medium	day 7	TIIV	0.00	0.00	0.00
9071	H1N1	day 7	TIIV	91.18	4.80	95.98
9071	SEB	day 7	TIIV	345.34	7.04	352.38
9071	medium	day 28	TIIV	0.00	0.00	0.00
9071	H1N1	day 28	TIIV	30.16	0.00	30.16
9071	SEB	day 28	TIIV	168.20	18.68	186.88
9083	medium	day 0	TIIV	0.00	0.00	0.00
9083	H1N1	day 0	TIIV	4.44	0.00	4.44
9083	SEB	day 0	TIIV	224.70	11.20	235.90
9083	medium	day 7	TIIV	0.00	0.00	0.00
9083	H1N1	day 7	TIIV	306.64	55.22	361.86
9083	SEB	day 7	TIIV	314.66	36.16	350.82
9083	medium	day 28	TIIV	0.00	0.00	0.00
9083	H1N1	day 28	TIIV	55.80	12.40	68.20
9083	SEB	day 28	TIIV	182.01	0.00	182.01
9004	Medium	day 0	TIIV	0.00	0.00	0.00
9004	H1N1	day 0	TIIV	24.20	0.00	24.20
9004	SEB	day 0	TIIV	364.80	0.00	364.80
9004	Medium	day 7	TIIV	0.00	0.00	0.00
9004	H1N1	day 7	TIIV	126.70	18.14	144.84
9004	SEB	day 7	TIIV	481.04	22.94	503.98
9004	Medium	day 28	TIIV	0.00	0.00	0.00
9004	H1N1	day 28	TIIV	72.11	9.01	81.12
9004	SEB	day 28	TIIV	214.50	0.00	214.50
9091	Medium	day 0	TIIV	0.00	0.00	0.00
9091	H1N1	day 0	TIIV	53.78	7.68	61.46
9091	SEB	day 0	TIIV	14.72	0.00	14.72
9091	Medium	day 7	TIIV	0.00	0.00	0.00
9091	H1N1	day 7	TIIV	5.87	5.87	11.74
9091	SEB	day 7	TIIV	19.33	6.43	25.76
9091	Medium	day 28	TIIV	0.00	0.00	0.00
9091	H1N1	day 28	TIIV	0.00	0.00	0.00
9091	SEB	day 28	TIIV	10.78	0.00	10.78
9073	Medium	day 0	ATIIV	0.00	0.00	0.00
9073	H1N1	day 0	ATIIV	18.80	9.42	28.22
9073	SEB	day 0	ATIIV	417.99	0.00	417.99

9073	Medium	day 7	ATIIV	0.00	0.00	0.00
9073	H1N1	day 7	ATIIV	205.28	115.72	321.00
9073	SEB	day 7	ATIIV	569.27	40.18	609.45
9073	Medium	day 28	ATIIV	0.00	0.00	0.00
9073	H1N1	day 28	ATIIV	45.63	7.61	53.24
9073	SEB	day 28	ATIIV	228.71	15.22	243.93
9078	Medium	day 0	ATIIV	0.00	0.00	0.00
9078	H1N1	day 0	ATIIV	10.50	0.00	10.50
9078	SEB	day 0	ATIIV	318.00	159.00	477.00
9078	Medium	day 7	ATIIV	0.00	0.00	0.00
9078	H1N1	day 7	ATIIV	114.92	0.00	114.92
9078	SEB	day 7	ATIIV	467.01	14.80	481.81
9078	Medium	day 28	ATIIV	0.00	0.00	0.00
9078	H1N1	day 28	ATIIV	23.80	0.00	23.80
9078	SEB	day 28	ATIIV	309.22	0.00	309.22
9010	medium	day 0	ATIIV	0.00	0.00	0.00
9010	H1N1	day 0	ATIIV	39.24	4.87	44.11
9010	SEB	day 0	ATIIV	0.00	0.00	0.00
9010	medium	day 7	ATIIV	0.00	0.00	0.00
9010	H1N1	day 7	ATIIV	174.24	10.88	185.12
9010	SEB	day 7	ATIIV	455.00	16.62	471.62
9010	medium	day 28	ATIIV	0.00	0.00	0.00
9010	H1N1	day 28	ATIIV	29.03	0.00	29.03
9010	SEB	day 28	ATIIV	99.85	0.00	99.85
9026	medium	day 0	ATIIV	0.00	0.00	0.00
9026	H1N1	day 0	ATIIV	77.06	3.31	80.37
9026	SEB	day 0	ATIIV	487.28	46.59	533.87
9026	medium	day 7	ATIIV	0.00	0.00	0.00
9026	H1N1	day 7	ATIIV	234.35	6.43	240.78
9026	SEB	day 7	ATIIV	1092.75	43.00	1135.75
9026	medium	day 28	ATIIV	0.00	0.00	0.00
9026	H1N1	day 28	ATIIV	211.90	11.45	223.35
9026	SEB	day 28	ATIIV	618.40	129.68	748.08
9031	MEDIUM	day 0	ATIIV	0.00	0.00	0.00
9031	H1N1	day 0	ATIIV	66.73	0.00	66.73
9031	SEB	day 0	ATIIV	190.76	8.54	199.30
9031	MEDIUM	day 7	ATIIV	0.00	0.00	0.00
9031	H1N1	day 7	ATIIV	128.44	24.19	152.63
9031	SEB	day 7	ATIIV	640.20	28.15	668.35
9031	MEDIUM	day 28	ATIIV	0.00	0.00	0.00
9031	H1N1	day 28	ATIIV	134.04	11.34	145.38
9031	SEB	day 28	ATIIV	300.14	26.70	326.84
9041	MEDIUM	day 0	ATIIV	0.00	0.00	0.00
9041	H1N1	day 0	ATIIV	215.50	19.85	235.35
9041	SEB	day 0	ATIIV	460.60	26.40	487.00
9041	MEDIUM	day 7	ATIIV	0.00	0.00	0.00
9041	H1N1	day 7	ATIIV	1489.29	33.86	1523.15
9041	SEB	day 7	ATIIV	401.76	37.48	439.24
9041	MEDIUM	day 28	ATIIV	0.00	0.00	0.00
9041	H1N1	day 28	ATIIV	506.17	23.85	530.02
9041	SEB	day 28	ATIIV	311.00	0.00	311.00
9043	MEDIUM	day 0	ATIIV	0.00	0.00	0.00
9043	H1N1	day 0	ATIIV	63.20	10.60	73.80
9043	SEB	day 0	ATIIV	1013.40	36.96	1050.36
9043	MEDIUM	day 7	ATIIV	0.00	0.00	0.00
9043	H1N1	day 7	ATIIV	266.59	56.33	322.92

9043	SEB	day 7	ATIIV	1370.09	138.20	1508.29
9043	MEDIUM	day 28	ATIIV	0.00	0.00	0.00
9043	H1N1	day 28	ATIIV	135.80	18.58	154.38
9043	SEB	day 28	ATIIV	1027.90	0.00	1027.90
9044	MEDIUM	day 0	ATIIV	0.00	0.00	0.00
9044	H1N1	day 0	ATIIV	124.80	31.20	156.00
9044	SEB	day 0	ATIIV	2114.70	177.10	2291.80
9044	MEDIUM	day 7	ATIIV	0.00	0.00	0.00
9044	H1N1	day 7	ATIIV	672.60	72.80	745.40
9044	SEB	day 7	ATIIV	1597.10	34.70	1631.80
9044	MEDIUM	day 28	ATIIV	0.00	0.00	0.00
9044	H1N1	day 28	ATIIV	188.50	14.00	202.50
9044	SEB	day 28	ATIIV	584.10	0.00	584.10
9046	MEDIUM	day 0	ATIIV	0.00	0.00	0.00
9046	H1N1	day 0	ATIIV	19.96	4.99	24.95
9046	SEB	day 0	ATIIV	238.10	18.30	256.40
9046	MEDIUM	day 7	ATIIV	0.00	0.00	0.00
9046	H1N1	day 7	ATIIV	28.15	0.00	28.15
9046	SEB	day 7	ATIIV	592.74	47.80	640.54
9046	MEDIUM	day 28	ATIIV	0.00	0.00	0.00
9046	H1N1	day 28	ATIIV	133.33	0.00	133.33
9046	SEB	day 28	ATIIV	297.00	0.00	297.00
9050	Medium	day 0	ATIIV	0.00	0.00	0.00
9050	H1N1	day 0	ATIIV	8.78	0.00	8.78
9050	SEB	day 0	ATIIV	92.60	4.87	97.47
9050	Medium	day 7	ATIIV	0.00	0.00	0.00
9050	H1N1	day 7	ATIIV	5.80	0.00	5.80
9050	SEB	day 7	ATIIV	50.11	0.00	50.11
9050	Medium	day 28	ATIIV	0.00	0.00	0.00
9050	H1N1	day 28	ATIIV	0.00	0.00	0.00
9050	SEB	day 28	ATIIV	33.85	0.00	33.85
9056	Medium	day 0	ATIIV	0.00	0.00	0.00
9056	H1N1	day 0	ATIIV	36.42	0.00	36.42
9056	SEB	day 0	ATIIV	344.50	20.30	364.80
9056	Medium	day 7	ATIIV	0.00	0.00	0.00
9056	H1N1	day 7	ATIIV	55.47	0.00	55.47
9056	SEB	day 7	ATIIV	592.70	31.20	623.90
9056	Medium	day 28	ATIIV	0.00	0.00	0.00
9056	H1N1	day 28	ATIIV	40.50	0.00	40.50
9056	SEB	day 28	ATIIV	1494.00	0.00	1494.00
9063	Medium	day 0	ATIIV	0.00	0.00	0.00
9063	H1N1	day 0	ATIIV	27.49	3.93	31.42
9063	SEB	day 0	ATIIV	192.37	12.27	204.64
9063	Medium	day 7	ATIIV	0.00	0.00	0.00
9063	H1N1	day 7	ATIIV	171.84	22.41	194.25
9063	SEB	day 7	ATIIV	434.00	0.00	434.00
9063	Medium	day 28	ATIIV	0.00	0.00	0.00
9063	H1N1	day 28	ATIIV	62.89	0.00	62.89
9063	SEB	day 28	ATIIV	254.80	0.00	254.80
9066	Medium	day 0	ATIIV	0.00	0.00	0.00
9066	H1N1	day 0	ATIIV	33.80	0.00	33.80
9066	SEB	day 0	ATIIV	286.68	9.88	296.56
9066	Medium	day 7	ATIIV	0.00	0.00	0.00
9066	H1N1	day 7	ATIIV	372.30	6.31	378.61
9066	SEB	day 7	ATIIV	1001.83	16.93	1018.76
9066	Medium	day 28	ATIIV	0.00	0.00	0.00

9066	H1N1	day 28	ATIIV	99.40	7.09	106.49
9066	SEB	day 28	ATIIV	937.80	20.10	957.90
9068	Medium	day 0	ATIIV	0.00	0.00	0.00
9068	H1N1	day 0	ATIIV	5.49	0.00	5.49
9068	SEB	day 0	ATIIV	103.68	0.00	103.68
9068	Medium	day 7	ATIIV	0.00	0.00	0.00
9068	H1N1	day 7	ATIIV	250.40	13.40	263.80
9068	SEB	day 7	ATIIV	434.90	24.50	459.40
9068	Medium	day 28	ATIIV	0.00	0.00	0.00
9068	H1N1	day 28	ATIIV	33.14	0.00	33.14
9068	SEB	day 28	ATIIV	205.87	5.41	211.28
9069	Medium	day 0	ATIIV	0.00	0.00	0.00
9069	H1N1	day 0	ATIIV	15.17	3.79	18.96
9069	SEB	day 0	ATIIV	549.27	12.24	561.51
9069	Medium	day 7	ATIIV	0.00	0.00	0.00
9069	H1N1	day 7	ATIIV	119.22	0.00	119.22
9069	SEB	day 7	ATIIV	172.42	0.00	172.42
9069	Medium	day 28	ATIIV	0.00	0.00	0.00
9069	H1N1	day 28	ATIIV	38.03	0.00	38.03
9069	SEB	day 28	ATIIV	757.98	18.70	776.68
9082	Medium	day 0	ATIIV	0.00	0.00	0.00
9082	H1N1	day 0	ATIIV	28.40	0.00	28.40
9082	SEB	day 0	ATIIV	1069.50	80.20	1149.70
9082	Medium	day 7	ATIIV	0.00	0.00	0.00
9082	H1N1	day 7	ATIIV	140.96	7.83	148.79
9082	SEB	day 7	ATIIV	929.40	44.98	974.38
9082	Medium	day 28	ATIIV	0.00	0.00	0.00
9082	H1N1	day 28	ATIIV	93.60	0.00	93.60
9082	SEB	day 28	ATIIV	1012.50	34.20	1046.70
9088	Medium	day 0	ATIIV	0.00	0.00	0.00
9088	H1N1	day 0	ATIIV	12.90	12.86	25.76
9088	SEB	day 0	ATIIV	1038.58	97.76	1136.34
9088	Medium	day 7	ATIIV	0.00	0.00	0.00
9088	H1N1	day 7	ATIIV	17.37	0.00	17.37
9088	SEB	day 7	ATIIV	295.10	5.78	300.88
9088	Medium	day 28	ATIIV	0.00	0.00	0.00
9088	H1N1	day 28	ATIIV	32.15	5.35	37.50
9088	SEB	day 28	ATIIV	1647.90	0.00	1647.90
9022	medium	day 0	Placebo	0.00	0.00	0.00
9022	H1N1	day 0	Placebo	0.00	0.00	0.00
9022	SEB	day 0	Placebo	222.90	11.40	234.30
9022	medium	day 7	Placebo	0.00	0.00	0.00
9022	H1N1	day 7	Placebo	0.00	0.00	0.00
9022	SEB	day 7	Placebo	146.10	24.40	170.50
9022	medium	day 28	Placebo	0.00	0.00	0.00
9022	H1N1	day 28	Placebo	0.00	0.00	0.00
9022	SEB	day 28	Placebo	79.20	11.30	90.50
9024	medium	day 0	Placebo	0.00	0.00	0.00
9024	H1N1	day 0	Placebo	15.36	0.00	15.36
9024	SEB	day 0	Placebo	554.80	7.20	562.00
9024	medium	day 7	Placebo	0.00	0.00	0.00
9024	H1N1	day 7	Placebo	18.95	0.00	18.95
9024	SEB	day 7	Placebo	443.71	0.00	443.71
9024	medium	day 28	Placebo	0.00	0.00	0.00
9024	H1N1	day 28	Placebo	12.50	6.26	18.76
9024	SEB	day 28	Placebo	284.20	18.15	302.35

9036	medium	day 0	Placebo	0.00	0.00	0.00
9036	H1N1	day 0	Placebo	11.40	11.40	22.80
9036	SEB	day 0	Placebo	312.00	78.00	390.00
9036	medium	day 7	Placebo	0.00	0.00	0.00
9036	H1N1	day 7	Placebo	76.93	10.26	87.19
9036	SEB	day 7	Placebo	605.10	94.00	699.10
9036	medium	day 28	Placebo	0.00	0.00	0.00
9036	H1N1	day 28	Placebo	5.20	5.20	10.40
9036	SEB	day 28	Placebo	305.50	0.00	305.50
9038	medium	day 0	Placebo	0.00	0.00	0.00
9038	H1N1	day 0	Placebo	182.40	12.20	194.60
9038	SEB	day 0	Placebo	797.68	25.08	822.76
9038	medium	day 7	Placebo	0.00	0.00	0.00
9038	H1N1	day 7	Placebo	97.74	26.64	124.38
9038	SEB	day 7	Placebo	1046.50	121.00	1167.50
9038	medium	day 28	Placebo	0.00	0.00	0.00
9038	H1N1	day 28	Placebo	159.48	0.00	159.48
9038	SEB	day 28	Placebo	344.10	0.00	344.10
9074	medium	day 0	Placebo	0.00	0.00	0.00
9074	H1N1	day 0	Placebo	25.30	0.00	25.30
9074	SEB	day 0	Placebo	507.56	8.06	515.62
9074	medium	day 7	Placebo	0.00	0.00	0.00
9074	H1N1	day 7	Placebo	7.24	7.24	14.48
9074	SEB	day 7	Placebo	526.31	86.51	612.82
9074	medium	day 28	Placebo	0.00	0.00	0.00
9074	H1N1	day 28	Placebo	27.84	6.97	34.81
9074	SEB	day 28	Placebo	896.91	99.61	996.52
9080	medium	day 0	Placebo	0.00	0.00	0.00
9080	H1N1	day 0	Placebo	52.32	0.59	52.91
9080	SEB	day 0	Placebo	1877.12	111.89	1989.01
9080	medium	day 7	Placebo	0.00	0.00	0.00
9080	H1N1	day 7	Placebo	37.50	0.00	37.50
9080	SEB	day 7	Placebo	1616.16	52.90	1669.06
9080	medium	day 28	Placebo	0.00	0.00	0.00
9080	H1N1	day 28	Placebo	40.80	6.82	47.62
9080	SEB	day 28	Placebo	1674.70	20.20	1694.90
9081	medium	day 0	Placebo	0.00	0.00	0.00
9081	H1N1	day 0	Placebo	49.87	0.00	49.87
9081	SEB	day 0	Placebo	582.80	28.20	611.00
9081	medium	day 7	Placebo	0.00	0.00	0.00
9081	H1N1	day 7	Placebo	11.81	7.66	19.47
9081	SEB	day 7	Placebo	936.36	95.82	1032.18
9081	medium	day 28	Placebo	0.00	0.00	0.00
9081	H1N1	day 28	Placebo	37.44	3.74	41.18
9081	SEB	day 28	Placebo	610.30	30.02	640.32
