# Supplementary material for: Early Rise of Blood T Follicular Helper Cell Subsets and Baseline Immunity as Predictors of Persisting Late Functional Antibody Responses to Vaccination in Humans
Source: PLoS One. 2016 Jun 23;11(6):e0157066. doi: 10.1371/journal.pone.0157066 (PMC4918887; doi:10.1371/journal.pone.0157066)
Supplement: S5 Table — (PDF) [file pone.0157066.s011.pdf]

S5 Table Frequency of Ex vivo CD4 T cells /10^6 PBMCs

SAMPLE ID	Vaccination	Visit	CD4+ICOS+CXCR5+	CD4+ICOS+CX5+CXCR3+	CD4+CX5+CXCR3+PD1+
9004	TIIV	Day 0	1702.70	391.89	2828.83
9004	TIIV	Day 7	7431.62	4188.03	4423.08
9004	TIIV	Day 28	940.74	207.41	1377.78
9017	TIIV	day 0	1662.39	256.41	871.79
9017	TIIV	day 7	3970.69	1746.55	1737.93
9017	TIIV	day 28	754.11	120.22	524.60
9018	TIIV	day 0	1086.14	344.57	1314.61
9018	TIIV	day 7	1579.44	588.79	1233.64
9018	TIIV	day 28	629.96	66.08	348.02
9021	TIIV	day 0	2351.17	1066.89	3120.40
9021	TIIV	day 7	2860.93	1665.56	2655.63
9021	TIIV	day 28	1782.61	663.04	2068.84
9027	TIIV	day 0	1242.90	397.48	1716.09
9027	TIIV	day 7	3334.48	2106.90	2700.00
9027	TIIV	day 28	691.63	277.53	1123.35
9028	TIIV	day 0	3223.12	754.56	3464.50
9028	TIIV	day 7	7882.35	3851.93	5630.83
9028	TIIV	day 28	1586.28	241.15	1727.88
9033	TIIV	day 0	759.09	433.76	2125.44
9033	TIIV	day 7	2970.27	2116.22	3335.14
9033	TIIV	day 28	537.19	74.38	347.11
9035	TIIV	day 0	3002.32	392.11	2062.65
9035	TIIV	day 7	2336.81	540.17	2090.21
9035	TIIV	day 28	1949.58	243.70	1462.18
9045	TIIV	day 0	789.16	228.92	807.23
9045	TIIV	day 7	4740.74	2703.70	3177.78
9045	TIIV	day 28	663.61	180.43	1244.65
9051	TIIV	day 0	852.86	179.84	2640.33
9051	TIIV	day 7	2097.92	941.67	2952.08
9051	TIIV	day 28	352.58	68.04	2492.78
9055	TIIV	day 0	942.78	133.51	1103.54
9055	TIIV	day 7	2514.14	1033.42	2321.34
9055	TIIV	day 28	987.62	321.78	2304.46
9057	TIIV	day 0	783.51	82.47	2036.08
9057	TIIV	day 7	1486.77	592.59	3211.64
9057	TIIV	day 28	895.03	66.30	2657.46
9061	TIIV	day 0	1464.37	457.00	2402.95
9061	TIIV	day 7	2025.39	822.27	2933.59
9061	TIIV	day 28	949.82	125.45	1232.97
9062	TIIV	day 0	1027.78	160.49	2117.28
9062	TIIV	day 7	2308.47	1417.34	3864.92
9062	TIIV	day 28	692.83	219.73	2069.51
9065	TIIV	day 0	637.50	18.75	768.75
9065	TIIV	day 7	3922.03	1555.93	3132.20
9065	TIIV	day 28	1410.26	261.54	1892.31
9071	TIIV	day 0	670.89	151.90	623.42
9071	TIIV	day 7	1819.35	948.39	1861.29
9071	TIIV	day 28	1212.87	361.39	1396.04
9083	TIIV	day 0	884.00	308.00	1728.00
9083	TIIV	day 7	6748.84	4702.33	5004.65
9083	TIIV	day 28	1811.00	814.43	2384.88
9091	TIIV	day 0	1025.08	128.53	1125.39
9091	TIIV	day 7	1956.24	621.44	1457.33
9091	TIIV	day 28	1356.29	154.39	1135.39

9010	ATIIV	day 0	630.37	48.71	567.34
9010	ATIIV	day 7	2084.21	915.79	1136.84
9010	ATIIV	day 28	383.05	47.46	325.42
9026	ATIIV	day 0	522.62	144.80	886.88
9026	ATIIV	day 7	972.97	509.01	1216.22
9026	ATIIV	day 28	507.85	99.48	560.21
9031	ATIIV	DAY 0	1332.32	210.37	1716.46
9031	ATIIV	DAY 7	2552.11	1270.42	2115.49
9031	ATIIV	DAY 28	1327.49	213.45	1590.64
9041	ATIIV	DAY 0	2054.69	382.81	2113.28
9041	ATIIV	DAY 7	10300.00	6809.09	6163.64
9041	ATIIV	DAY 28	1383.42	264.25	1823.83
9043	ATIIV	DAY 0	1315.79	240.60	2411.03
9043	ATIIV	DAY 7	15280.19	8574.88	7545.89
9043	ATIIV	DAY 28	1374.67	234.83	1263.85
9044	ATIIV	DAY 0	1653.98	391.00	2083.04
9044	ATIIV	DAY 7	16094.09	10557.99	7752.74
9044	ATIIV	DAY 28	1133.90	455.84	1752.14
9046	ATIIV	DAY 0	872.61	140.13	1181.53
9046	ATIIV	DAY 7	1075.95	488.05	853.73
9046	ATIIV	DAY 28	554.43	88.61	787.34
9050	ATIIV	day 0	761.57	164.35	1652.78
9050	ATIIV	day 7	590.12	191.86	970.93
9050	ATIIV	day 28	318.01	53.64	452.11
9056	ATIIV	day 0	405.20	85.50	394.05
9056	ATIIV	day 7	902.61	166.27	451.31
9056	ATIIV	day 28	161.46	10.42	41.67
9063	ATIIV	day 0	808.76	201.20	1826.69
9063	ATIIV	day 7	3613.29	2364.57	3904.60
9063	ATIIV	day 28	853.29	155.69	1628.74
9066	ATIIV	day 0	380.95	80.59	1545.79
9066	ATIIV	day 7	1125.41	561.06	3544.55
9066	ATIIV	day 28	741.38	178.16	2310.34
9068	ATIIV	day 0	845.03	187.13	4239.77
9068	ATIIV	day 7	11556.49	7569.04	9000.00
9068	ATIIV	day 28	1440.37	353.21	3811.93
9069	ATIIV	day 0	1732.56	207.36	2955.43
9069	ATIIV	day 7	2834.08	789.24	1206.28
9069	ATIIV	day 28	2613.64	439.39	2962.12
9073	ATIIV	day 0	1127.45	411.76	1887.25
9073	ATIIV	day 7	8479.77	5352.60	6716.76
9073	ATIIV	day 28	1061.58	410.56	2577.71
9078	ATIIV	day 0	635.90	51.28	512.82
9078	ATIIV	day 7	1867.47	144.58	213.86
9078	ATIIV	day 28	813.29	101.27	895.57
9082	ATIIV	Day 0	671.79	143.59	897.44
9082	ATIIV	Day 7	4231.25	2550.00	3131.25
9082	ATIIV	Day 28	1032.11	192.66	1261.47
9088	ATIIV	Day 0	2307.89	955.26	3065.79
9088	ATIIV	Day 7	1790.18	908.48	3229.91
9088	ATIIV	Day 28	1289.59	538.46	2298.64
9022	Placebo	DAY 0	1266.22	290.83	1476.51
9022	Placebo	DAY 7	1830.51	474.58	1847.46
9022	Placebo	DAY 28	1279.79	191.71	1222.80
9024	Placebo	day 0	1408.70	86.96	817.39
9024	Placebo	day 7	1496.77	64.52	948.39

9024	Placebo	day 28	1439.81	97.22	768.52
9036	Placebo	day 0	2547.17	566.04	2477.09
9036	Placebo	day 7	2697.14	522.86	2408.57
9036	Placebo	day 28	1813.90	255.61	2334.08
9038	Placebo	day 0	1810.81	324.32	864.86
9038	Placebo	day 7	1474.79	205.88	567.23
9038	Placebo	day 28	421.49	24.79	305.79
9074	Placebo	day 0	844.09	107.53	876.34
9074	Placebo	day 7	1162.16	123.55	1563.71
9074	Placebo	day 28	1628.00	280.00	1644.00
9080	Placebo	day 0	1134.33	288.56	2920.40
9080	Placebo	day 7	1088.40	251.38	2306.63
9080	Placebo	day 28	1909.76	560.98	2848.78
9081	Placebo	day 0	650.15	126.93	1718.27
9081	Placebo	day 7	1117.85	262.63	2074.07
9081	Placebo	day 28	1143.22	243.72	1472.36
